# Identification of Anticancer Enzymes and Biomarkers for Hepatocellular Carcinoma through Constraint-Based Modeling

**DOI:** 10.3390/molecules29112594

**Published:** 2024-05-31

**Authors:** Feng-Sheng Wang, Hao-Xiang Zhang

**Affiliations:** Department of Chemical Engineering, National Chung Cheng University, Chiayi 621301, Taiwan; aa0970797987@gmail.com

**Keywords:** parsimonious flux balance analysis, parsimonious flux variability analysis, genome-scale metabolic model, fuzzy hierarchical optimization, hybrid differential evolution, drug target discovery

## Abstract

Hepatocellular carcinoma (HCC) results in the abnormal regulation of cellular metabolic pathways. Constraint-based modeling approaches can be utilized to dissect metabolic reprogramming, enabling the identification of biomarkers and anticancer targets for diagnosis and treatment. In this study, two genome-scale metabolic models (GSMMs) were reconstructed by employing RNA sequencing expression patterns of hepatocellular carcinoma (HCC) and their healthy counterparts. An anticancer target discovery (ACTD) framework was integrated with the two models to identify HCC targets for anticancer treatment. The ACTD framework encompassed four fuzzy objectives to assess both the suppression of cancer cell growth and the minimization of side effects during treatment. The composition of a nutrient may significantly affect target identification. Within the ACTD framework, ten distinct nutrient media were utilized to assess nutrient uptake for identifying potential anticancer enzymes. The findings revealed the successful identification of target enzymes within the cholesterol biosynthetic pathway using a cholesterol-free cell culture medium. Conversely, target enzymes in the cholesterol biosynthetic pathway were not identified when the nutrient uptake included a cholesterol component. Moreover, the enzymes PGS1 and CRL1 were detected in all ten nutrient media. Additionally, the ACTD framework comprises dual-group representations of target combinations, pairing a single-target enzyme with an additional nutrient uptake reaction. Additionally, the enzymes PGS1 and CRL1 were identified across the ten-nutrient media. Furthermore, the ACTD framework encompasses two-group representations of target combinations involving the pairing of a single-target enzyme with an additional nutrient uptake reaction. Computational analysis unveiled that cell viability for all dual-target combinations exceeded that of their respective single-target enzymes. Consequently, integrating a target enzyme while adjusting an additional exchange reaction could efficiently mitigate cell proliferation rates and ATP production in the treated cancer cells. Nevertheless, most dual-target combinations led to lower side effects in contrast to their single-target counterparts. Additionally, differential expression of metabolites between cancer cells and their healthy counterparts were assessed via parsimonious flux variability analysis employing the GSMMs to pinpoint potential biomarkers. The variabilities of the fluxes and metabolite flow rates in cancer and healthy cells were classified into seven categories. Accordingly, two secretions and thirteen uptakes (including eight essential amino acids and two conditionally essential amino acids) were identified as potential biomarkers. The findings of this study indicated that cancer cells exhibit a higher uptake of amino acids compared with their healthy counterparts.

## 1. Introduction

The liver is a pivotal metabolic organ that processes and transforms nutrients, stores energy, and synthesizes essential molecules. Hepatocytes, constituting approximately 85% of the liver’s total mass, perform the majority of these metabolic processes [1]. Hepatocellular carcinoma (HCC) is a liver disease wherein numerous metabolic processes are dysregulated, fostering tumorigenesis [2]. Globally, HCC ranks as the third-leading cause of cancer-related mortality [3,4]. The dysregulation of liver metabolism alters cellular metabolic pathways and is associated with cancer cells that appear different from normal cells [5]. Similar to other cancer cells, HCC cells undergo metabolic changes, including aerobic glycolysis, diminished oxidative phosphorylation, and heightened biosynthetic intermediate generation to sustain rapid growth and proliferation [6,7,8,9,10].

Numerous studies have leveraged metabolic characteristics and reprogramming to comprehend cancer-specific metabolic networks, identify potential anticancer targets, and, thus, inhibit tumor growth or viability [10,11,12,13,14,15,16,17,18,19,20,21,22,23,24]. Many drug discovery strategies, as noted in studies [11,12,13,14,15,16,17,18,19], have employed cancer cell mortality as a metric to gauge the effectiveness of the identified targets. However, the drug development process poses significant challenges due to its high expenses and prolonged timelines. A major hurdle is the efficient identification of both drug targets and potential side effects. Traditionally, side effects manifest primarily during pre-clinical or clinical trials, allowing for assessment of their severity. However, there is a notable lack of research focusing on predicting candidate target side effects in the early stages. In this study, we propose the introduction of a side effect index for target discovery procedures. This index aims to assess deviations between metabolic patterns of perturbed healthy cells in comparison to the respective cancer and healthy templates.

Tissue-specific genome-scale metabolic models (GSMMs) have been reconstructed through constraint-based modeling approaches to identify anticancer targets for treatment [13,15,17,18,19,20,21,22,23,24,25,26,27,28] and predict biomarkers for diagnosis [16,29,30,31]. Synthetic lethality frequently serves as a therapeutic index in screening potential targets [25,28]. Notably, few studies have attempted to predict the side effects of the identified targets during the early stages of drug discovery. Considering that the dysregulation of human cells can lead to metabolic perturbations or alterations in healthy cells, the present study used the cell mortality index to identify anticancer targets while accounting for metabolic perturbations in healthy cells to predict side effects during target discovery. However, such approaches lack prediction of metabolic perturbations for each identified target. Fuzzy similarity and fuzzy dissimilarity have been introduced as indicators of metabolic perturbations for use in predicting the side effects associated with the identified targets [24]. Moreover, parsimonious flux variability analysis [32] was applied to evaluate the differential expression of metabolites between cancer cells and their healthy cells to identify potential biomarkers.

## 2. Results and Discussion

### 2.1. Weighting Factors in the UFD Problem

We extracted RNA-Seq expression data from 50 healthy liver samples with FPKM-UQ normalized data and 371 hepatocellular carcinoma samples, each with a distinct TCGA barcode, from the TCGA database [33]. These data were used to reconstruct tissue-specific GSMMs for healthy (HT) and cancer (CA) states using the CORDA algorithm [34]. The HT model comprised 4154 metabolites, 6652 reactions, and 2143 gene-encoding enzymes, whereas the CA model comprised 3982 metabolites, 6561 reactions, and 2107 gene-encoding enzymes. The GSMMs for the CA and HT models, stored in Excel format, are available in Supplementary File S1.

The Warburg effect, a common occurrence in the metabolic reprogramming of cancer cells, which involves an increase in the rate of glucose uptake and preferential production of lactate, even in the presence of oxygen [35], was observed, leading to the hypothesis that it could increase or decrease the production rate of metabolites [35,36,37]. We examined the trends in flux changes based on the Warburg hypothesis found in the literature [6,8,20,37] and compared them with the computational results obtained for the pFBA problems using Equation (1). As expressed by Equation (2), quartile weighting factors were employed to solve the pFBA problems and calculate the flux distributions for both the CA and HT models. This approach led to a remarkable overlap of approximately 63.9% when compared with the flux change trends derived from the Warburg hypothesis. Additionally, pFBA evaluations using equal weightings (i.e., $w_{j}^{CA/HT}=1$ for all reactions) resulted in a similarity of 59.1% with the trends observed in the Warburg hypothesis.

### 2.2. Single Anticancer Targets

We used Dulbecco’s Modified Eagle Medium (DMEM), exhibiting 54 uptake reactions that were configured as reversible and exchangeable reactions, as illustrated in Supplementary File S1. Secretion reactions in the GSMM were configured as irreversible reactions. The NHDE algorithm [38] was applied to solve the MDM problem, to identify anticancer target genes. The algorithm can be used to identify all conceivable single-target anticancer enzymes and suppress biomass growth in HCC. Multiple iterations resulted in the identification of 22 single-target enzymes (refer to Table 1) from a pool of 2034 candidate enzymes. Downregulating these enzymes can suppress the biomass growth rate of cancer cells from 4% to 100%. The identified enzymes—ADSL, ADSS2, GK, CTSA*, and GMPR2—in Table 1 represent duplicate enzymes in GSMMs. CTSA* is a complex enzyme consisting of the four genes CTSA, GLB1, NEU1, and PSAP.

**Figure 1:**
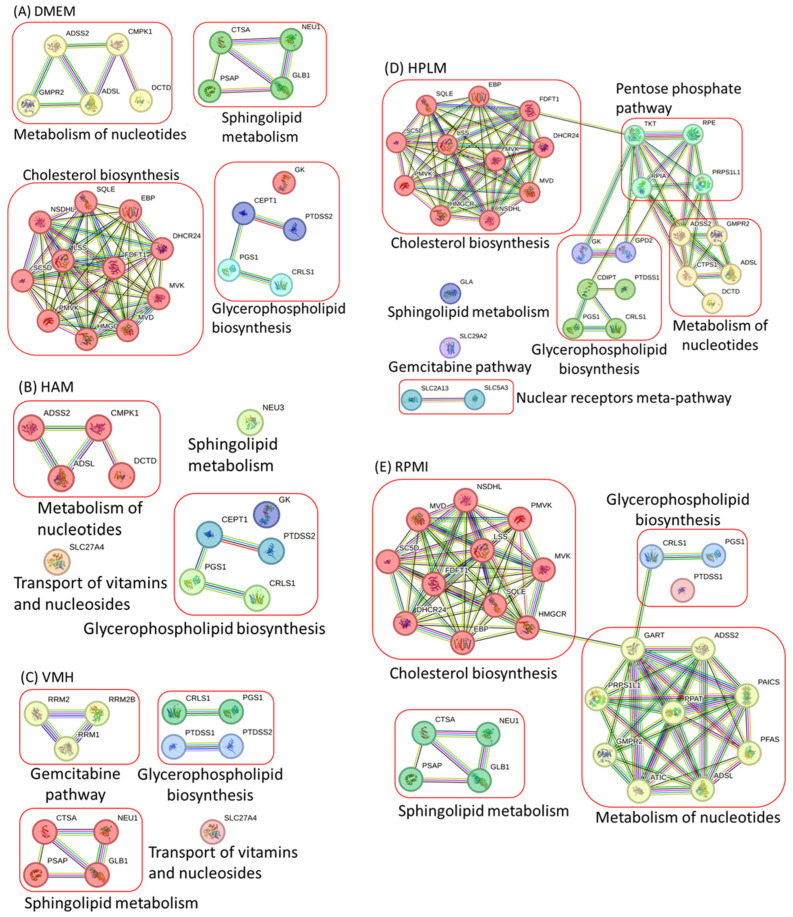
Protein–protein interactions of anticancer target enzymes in various nutrient media. (**A**) DMEM: Dulbecco’s Modified Eagle Medium. (**B**) HAM: Ham’s medium. (**C**) VMH: Uptake reactions obtained from the VMH database (https://www.vmh.life/#home accessed on 10 April 2023). (**D**) HPLM: Human plasma-like medium. (**E**) RPMI: Roswell Park Memorial Institute.

The ACTD framework was employed to identify overall single-target genes and examine the protein–protein interaction (PPI) network based on the STRING (https://string-db.org/, accessed on 21 August 2023) and GeneCards (https://www.genecards.org/, accessed on 21 August 2023) databases. Subsequently, the network was divided into four distinct classes, as depicted in Figure 1A. The first class comprised eleven genes involved in cholesterol biosynthesis; the second class comprised five genes involved in nucleotide metabolism; the third class comprised five genes involved in glycerophospholipid biosynthesis; and the fourth class comprised four genes involved in sphingolipid metabolism. These targets enhanced the cancer cell elimination rate from 4% to 100% and reduced the maximum ATP synthesis rate of 38 mmol/gDW/h to 6.618 mmol/gDW/h for TR cells. The corresponding cell viability grade ($\eta_{CV}^{TR}$) values ranged from 0.224 to 0.869. The metabolic deviation grade ($\eta_{MD}^{TP})$ for a treatment, an indicator of side effects, can be calculated based on the fuzzy similarity and fuzzy dissimilarity of the metabolic patterns for TR cells and PH cells relative to the metabolic templates of the HT and CA cells. The metabolic deviation grade values ranged from 0.269 to 0.479.

As indicated by the data in Table 1, the highest cell viability grade ($\eta_{CV}^{TR}$) of 0.896 was obtained for the respective treatment for the knockout of cytidine/uridine monophosphate kinase 1 (CMPK1) or downregulation of dCMP deaminase (DCTD). The metabolic deviation grade was 0.472 for the CMPK1 treatment and 0.451 for DCTD, respectively. Thus, CMPK1 induces fewer side effects than DCTD. The enzyme CMPK1 is involved in nucleotide metabolism, specifically in the phosphorylation of cytidine monophosphate (CMP) to form cytidine diphosphate (CDP). DCTD catalyzes the conversion of deoxycytidine monophosphate (dCMP) into deoxyuridine monophosphate (dUMP) by removing the amino group (NH2) from the cytosine base of dCMP. Both enzymes play a crucial role in nucleotide metabolism and are indirectly associated with the Gemcitabine pathway. Gemcitabine is a chemotherapeutic drug commonly used to treat various cancers. We examined DrugBank [39] to determine the number of drugs approved for the treatment of human diseases using the enzymes identified in Table 1. Nine drugs acting on CMPK1 and one on DCTD emerged as potential candidates for drug repurposing and treating HCC.

### 2.3. Various Nutrient Media

Ham’s medium was employed as the nutrient source, with 68 uptake reactions (see Supplementary File S1) employed to identify anticancer targets for treating the CA model. Ten target genes were identified using the ACTD framework, as illustrated in Table 1. The computational results indicated that the cell viability grades obtained using Ham’s medium were nearly equivalent to those derived from DMEM. However, the metabolic deviation grades with Ham’s medium were slightly higher than those with DMEM. 

The PPI network of the identified genes is depicted in Figure 1B. A comparison of Figure 1A,B reveals that enzymes associated with nucleotide metabolism, glycerophospholipid biosynthesis, sphingolipid metabolism, and the transport of vitamins and nucleotides reduced the biomass growth rate of treated cancer cells in Ham’s medium. However, enzymes linked to cholesterol biosynthesis did not suppress the biomass growth (Table 1) and, thus, were not associated with the identified enzymes (Figure 1B). 

The biomass growth suppression by an enzyme involved in cholesterol biosynthesis depends on whether the medium includes cholesterol uptake. To explore this further, we extended our analysis to three different media: VMH medium, human plasma-like medium (HPLM), and Roswell Park Memorial Institute (RPMI) medium. The uptake reactions in each medium are illustrated in Supplementary File S1. The VMH medium, sourced from the VMH database (https://www.vmh.life/#home accessed on 10 April 2023), was utilized to simulate human nutrient uptake. The ACTD framework identified potential single-target enzymes for the three media, with Table 1 summarizing the identified enzymes for each medium. The PPI network of the identified enzymes for these three media is illustrated in Figure 1C,D. Notably, the enzymes involved in cholesterol biosynthesis were unidentifiable using the VMH medium.

**Figure 2:**
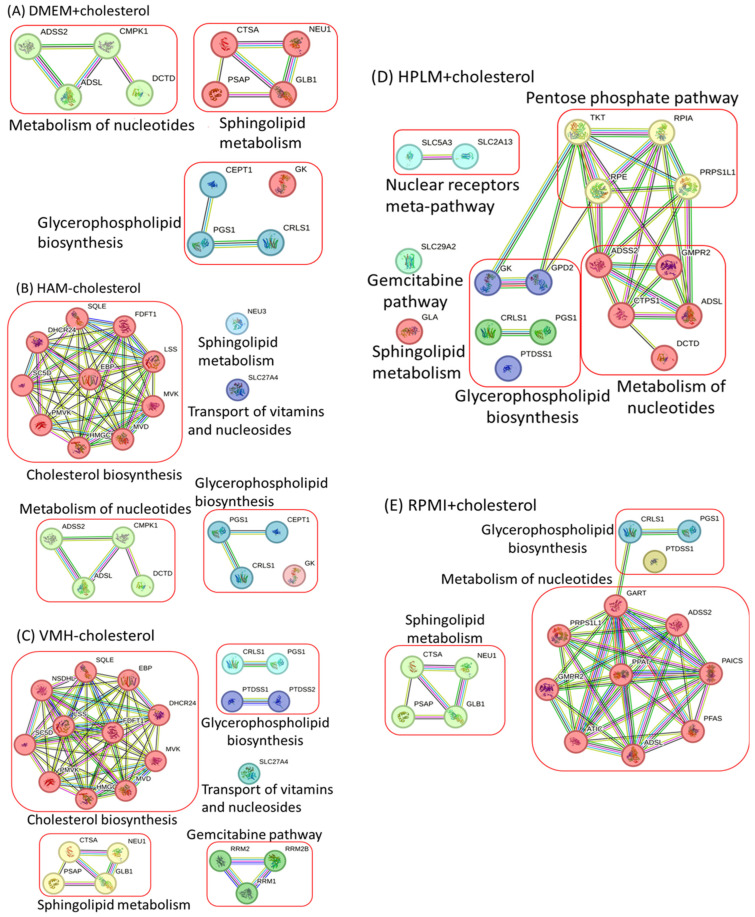
Protein–protein interactions of anticancer target enzymes using various nutrient media with or without cholesterol uptake. (**A**) DMEM+cholesterol: DMEM included additional cholesterol uptake. (**B**) HAM−cholesterol: HAM excluded cholesterol uptake from the medium. (**C**) VMH−cholesterol: VMH excluded cholesterol uptake from the medium. (**D**) HPLM+cholesterol: HPLM included additional cholesterol uptake. (**E**) RPMI+cholesterol: RPMI included additional cholesterol uptake.

A comparison of nutrient uptake reactions in five different nutrient media revealed the absence of the cholesterol uptake reaction in DMEM, HPLM, and RPMI. We used five additional media to assess nutrient uptake: DMEM, HPLM, and RPMI, which exhibited the cholesterol uptake reaction and were denoted as DMEM+cholesterol, HPLM+cholesterol, and RPMI+cholesterol, respectively. In contrast, Ham and VMH were employed without the cholesterol uptake reaction, denoted as Ham−cholesterol and VMH−cholesterol, respectively. The ACTD framework was applied to identify single anticancer targets and investigate the relationships between biomass growth and different nutrient components across these nutrient media. The ACTD framework identified all potential single-target enzymes (Supplementary File S2) for the ten nutrient media. Table 2 lists the enzymes identified for each medium. The PPI networks of the identified enzymes for these media are depicted in Figure 2A–D. Our analysis revealed that anticancer enzymes involved in cholesterol biosynthesis were identifiable when cholesterol uptake did not occur in a medium, as illustrated in Figure 1A,D,E and Figure 2B,C. Conversely, when the cholesterol uptake reaction occurred in the medium, the anticancer genes involved in cholesterol biosynthesis became unidentifiable, as illustrated in Figure 1B,C and Figure 2A,D,E.

### 2.4. Combination of Targets with Exchange Reactions

We incorporated a two-group representation within the ACTD framework to identify potential target combinations for treating HCC. Some of the single-target genes listed in Table 1 and Table 2 could not reduce the biomass growth rate of TR cells satisfactorily. Therefore, a two-group approach was employed to identify target combinations, pairing a single-target gene from Table 1 with an additional exchange reaction. Numerous combinations were identified using various nutrient media, with or without the cholesterol uptake reaction, as detailed in Supplementary File S3. Table 3 presents select combinations using DMEM, Ham’s, and VMH media from Supplementary File S3. Computational results revealed that the cell viability grades for all two-target combinations exceeded those for their corresponding one-target enzymes. Hence, combining a target enzyme with the modulation of an additional exchange reaction effectively reduced the biomass growth rate and ATP production rate in the treated cancer cells. Moreover, most two-target combinations exhibited an enhanced metabolic deviation grade relative to their corresponding single-target enzymes.

The cholesterol uptake reaction is pivotal in both Ham’s and VMH media and renders single-target enzymes involved in the cholesterol pathway inadequate for treating HCC. However, incorporating one of these enzymes in an additional exchange reaction in these media can suppress the biomass growth of TR cells, thereby achieving a cell viability grade of 0.842 in Ham’s medium and 0.980 in the VMH medium, as illustrated in Table 3. Thus, enabling an additional exchange reaction using a target enzyme can substantially enhance the therapeutic outcomes while minimizing the metabolic disturbances. As illustrated in Supplementary File S3, some combinations require at least two exchange reactions to improve the therapeutic outcomes, for example, knockout of NSDHL combined with two additional exchange reactions, as illustrated in Table 3.

### 2.5. Biomarker Identification

**Figure 3:**
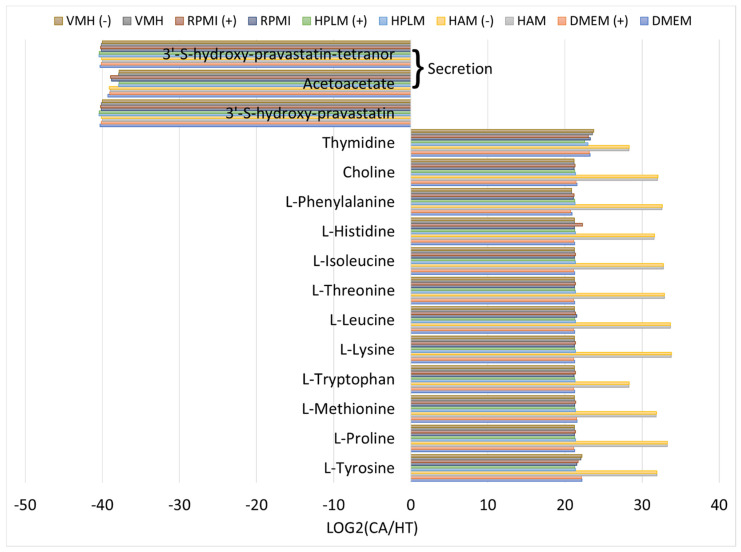
Log2 fold changes of exchange reactions between CA and HT cells. The minimum and maximum flux values for each exchange reaction in CA and HT cells were entirely increased or decreased, and the identified biomarkers were shared for ten nutrient media. “(+)” indicates that a nutrient medium included additional cholesterol uptake. “(−)” indicates that a nutrient medium excluded cholesterol uptake.

**Figure 4:**
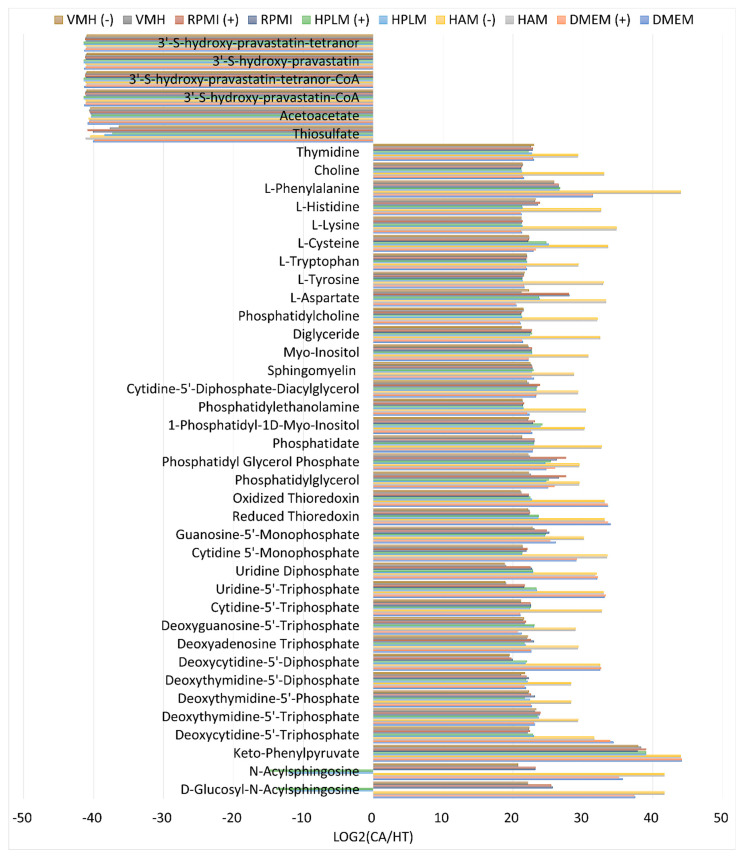
Log2 fold changes of metabolite flow rates between CA and HT cells. The minimum and maximum synthesis rates for each metabolite reaction in CA and HT cells were entirely increased or decreased, and the identified biomarkers were shared for ten nutrient media, except N-acylsphingosine and D-glucosyl-N-acylsphingosine. “(+)” indicates that a nutrient medium included additional cholesterol uptake. “(−)” indicates that a nutrient medium excluded cholesterol uptake.

We evaluated the flux distributions of the pFVA problem for the CA and HT models using ten nutrient media. Each nutrient medium yielded corresponding metabolite differential expression patterns between the CA and HT models. These patterns were used to calculate the mean of the distributions and determine the minimum and maximum values of secretion, uptake fluxes, and metabolite flow rates for the CA and HT models. The metabolites with differential expression for the CA and HT models with a log2 fold change greater than two are illustrated in Supplementary Files S4 and S5. Subsequently, potential biomarkers were identified using categories of “complete increase” or “complete decrease”. However, some identified biomarkers depended on the nutrient medium used. We compiled the identified biomarkers shared across ten nutrient media, as illustrated in Figure 3 and Figure 4. In the cancer state illustrated in Supplementary File S4, the secretion fluxes of 3′-S-hydroxy-pravastatin-tetranor and acetoacetate were zero, indicating a significant decrease relative to those in the healthy state (Figure 3). The uptake of 3′-S-hydroxy-pravastatin significantly decreased, whereas that of the others increased. According to our findings, two secretions and thirteen uptakes could serve as potential biomarkers. The uptakes included eight essential amino acids and two conditional essential amino acids, indicating that cancer cells exhibit higher uptake rates toward amino acids compared with healthy cells.

**Figure 5:**
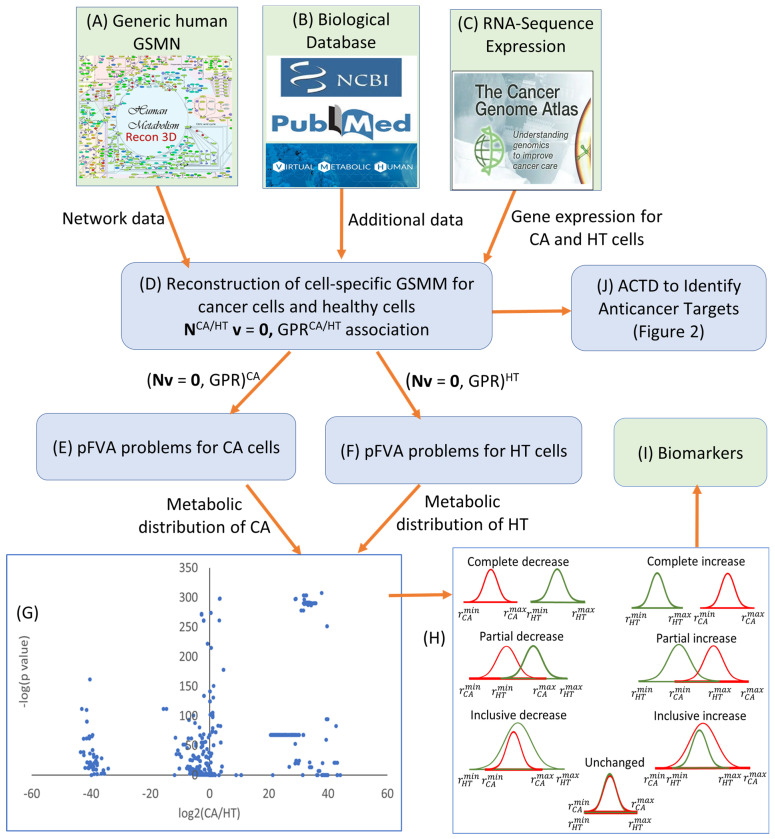
Workflow for identifying biomarkers using differential expression patterns between CA and HT models through pFVA problems. (**A**) Use of a generic human genome-scale metabolic network to reconstruct tissue-specific metabolic models. (**B**) Utilization of biological databases to specify the context of nutrients and pathways. (**C**) Incorporation of RNA-sequence expression patterns of cancer cells and their healthy counterparts for reconstructing tissue-specific metabolic models. (**D**) Reconstruction of tissue-specific GSMMs and gene-protein-reaction models for CA cells and HT cells. (**E**) Formulation of the pFVA problem for the CA model to compute a set of flux distributions. (**F**) Formulation of the pFVA problem for the HT model to compute a set of flux distributions. (**G**) Computation of log2(CA/HT) with respect to the *p*-values. (**H**) Normal or Gaussian distributions are categorized into seven distinct groups. The red curve indicates the distributions in the cancer state, and the green curve indicates that of the healthy state. $r_{CA/HT}^{max}$ and $r_{CA/HT}^{min}$ denote the maximum and minimum levels for each flux and metabolite flow rate in the cancer and healthy state. (**I**) Groups in “complete decrease” or “complete increase” are identified as potential biomarkers. (**J**) The reconstructed GSMMs are used for the ACTD framework to identify the anticancer targets discussed in Figure 6.

Figure 4 illustrates the log2-fold changes in metabolite flow rates between CA and HT cells. The metabolite flow rate, as defined in Equation (4), corresponds to the overall synthesis rate of the *m*th metabolite in the GSMM. As illustrated in the classification of Figure 5, six metabolites exhibited a complete decrease. Among these metabolites, 3′-S-hydroxy-pravastatin-tetranor, 3′-S-hydroxy-pravastatin, and acetoacetate were consistent with those identified in Figure 3. Additionally, 34 metabolites exhibited elevated levels, with thymidine, choline, and six amino acids among them exhibiting patterns similar to the uptake reactions depicted in Figure 3. Computational results indicated that the log2 fold changes of N-acylsphingosine and D-glucosyl-N-acylsphingosine between CA and HT cells were completely decreased using HPLM regardless of the occurrence or absence of the cholesterol uptake reaction; however, the levels of the other enzymes were elevated. Thus, N-acylsphingosine and D-glucosyl-N-acylsphingosine were influenced by the specific medium employed and, thus, cannot be considered candidate biomarkers.

## 3. Methods

### 3.1. Parsimonious Flux Balance Analysis and Flux Variability Analysis

The metabolic processes of HCC and its healthy counterpart were modeled through a constraint-based modeling approach to formulate it as a parsimonious flux balance analysis (pFBA) problem, which is an extension of flux balance analysis (FBA). In this modeling technique, the most efficient and minimal flux distribution through a metabolic network is determined to achieve a given objective. Notably, pFBA encompasses an FBA problem and a uniform flux distribution (UFD) problem, as follows:

(1) $$\left\{\begin{array}{l} \left\{\begin{array}{l} \mathrm{FBA}\;\mathrm{problem}:\\ \underset{v_{f},v_{b}}{\max}\;obj=c^{T,CA/HT}\left(v_{f}-v_{b}\right)\\ \mathrm{subject}\;\mathrm{to}\\ N^{CA/HT}\left(v_{f}-v_{b}\right)=0\\ v_{f/b,i}^{LB}\leq v_{f/b,i}\leq v_{f/b,i}^{UB},\;i\in \Omega \end{array}\right\}\\ \left\{\begin{array}{l} \mathrm{UFD}\;\mathrm{problem}:\\ \underset{v_{f},v_{b}}{\min}\sum_{k\in \Omega^{Int}}w_{j}^{CA/HT}\left(v_{f,j}+v_{b,j}\right)\\ \mathrm{subject}\;\mathrm{to}\\ N^{CA/HT}\left(v_{f}-v_{b}\right)=0\\ v_{f/b,i}^{LB}\leq v_{f/b,i}\leq v_{f/b,i}^{UB},\;i\in \Omega \;\\ obj\geq \zeta obj^{*} \end{array}\right\} \end{array}\right.$$

where **N***^CA/HT^* represents the stochiometric matrices for the cancer (CA) and healthy (HT) models reconstructed based on a generic human network (Recon3D) [40] using RNA-Seq expression data from The Cancer Genome Atlas (TCGA) database [33]. The parameters $v_{f/b,i}^{LB}$ and $v_{f/b,i}^{UB}$ represent the positive lower bound and positive upper bound of the *i*th forward flux and *i*th backward flux, respectively. The row vector **c***^T^*^,*CA/HT*^ contains raw weights indicating each reaction’s contribution to the objective function for the CA and HT models. The weighting factors $w_{j}^{CA/HT}$ in the UFD problem depend on confidence scores derived from gene–protein–reaction (GPR) associations. obj* is the maximal objective value obtained from the FBA problem, and the parameter ζ is assigned a value of 1 for pFBA. All reactions are classified using RNA-Seq expression of CA cells and HT cells and the GPR association of Recon3D into four types of confidence groups and can be assigned as follows:

(2) $$w_{j}^{CA/HT}=\left\{\begin{array}{l} 1/4,\;j\in \mathrm{high}\;\mathrm{confidence}\\ 1/2,\;j\in \mathrm{medium}\;\mathrm{confidence}\\ 3/4,\;j\in \mathrm{negativec}\;\mathrm{confidence}\\ 1,\;j\in \mathrm{other}\;\mathrm{confidence}\;\mathrm{or}\;\mathrm{non-gene-expression} \end{array}\right.$$

A small weighting factor indicates that a higher flux value in the UFD problem can be achieved when the corresponding reaction has a high confidence score in the GPR association.

The optimal fluxes for the pFBA problem are obtained with the fixed parameter ζ = 1, a value that can generally vary between 0 and 1. To account for this variability, we introduced parsimonious flux variability analysis (pFVA), which involves evaluating the metabolite differential expressions between CA and HT cells. The pFVA formulation is expressed as follows:

(3) $$\left\{\begin{array}{c} \underset{\zeta }{\max}/min\;r_{m}\\ \mathrm{subject}\;\mathrm{to}\;\\ \;\mathrm{FBA}\;\mathrm{problem}\\ \;\mathrm{UFD}\;\mathrm{problem} \end{array}\right.$$

where *r_m_* is the *m*th metabolite flow rate, and is expressed as follows:

(4) $$r_{m}=\sum_{m_{i}\in \Omega^{c}}\left(\sum_{N_{m_{i},j}>0,j}N_{m_{i},j}v_{f,j}-\sum_{N_{m_{i},j}<0,j}N_{m_{i},j}v_{b,j}\right),m\in \Omega^{m}$$

where Ω*^c^* is a set of metabolites located in various compartments of the cell and $N_{m_{i},j}$ is the stoichiometric coefficient of the *m*th metabolite at the *i*th compartment participating in the *j*th reaction in a GSMM. The overall synthesis rates for the *m*th metabolite in various compartments are summed to yield the *m*th metabolite flow rate.

Figure 5 illustrates a workflow using pFVA problems to calculate distributions for fluxes and metabolite flow rates toward achieving potential biomarkers. This workflow comprises two frameworks: one for cell-specific metabolic network reconstruction and another for biomarker identification. The initial framework involves employing reconstruction methods like CORDA [34] or iMAT [41] to reconstruct cell-specific GSMMs for CA and HT cells, as depicted in Figure 5A–D. The procedures for reconstruction are detailed in the literature [21,22,23,24]. Both models are used to establish the pFVA problems to yield distributions for the flux values and metabolite flow rates for CA and HT cells (Figure 5E,F). The computational data are utilized to calculate the corresponding normal or Gaussian distributions for cancer and healthy cells. 

As depicted in Figure 5G, a volcano plot illustrates the relationship between log2 fold change and their *p*-values. Biomarkers are commonly identified using log2 fold change and a *p*-value less than 0.05. However, this approach may yield numerous biomarkers, and determining clinical relevance becomes challenging when they fall within the overlap region of the distributions, making it difficult to discern their association with cancer or a healthy state. Utilizing these distributions, we established the minimum and maximum levels for each flux and metabolite flow rate in both cancer and healthy states. In this study, the flux variances shown in Figure 5G can be subsequently categorized into seven distinct groups, as shown in Figure 5H. A potential biomarker can be identified through comparative analysis: a flux value falling into the “complete increase” or “complete decrease” category indicates a significant difference in flux and metabolite flow rate between the cancer and healthy states, with no overlap in variability.

### 3.2. Anticancer Target Discovery Problem

**Figure 6:**
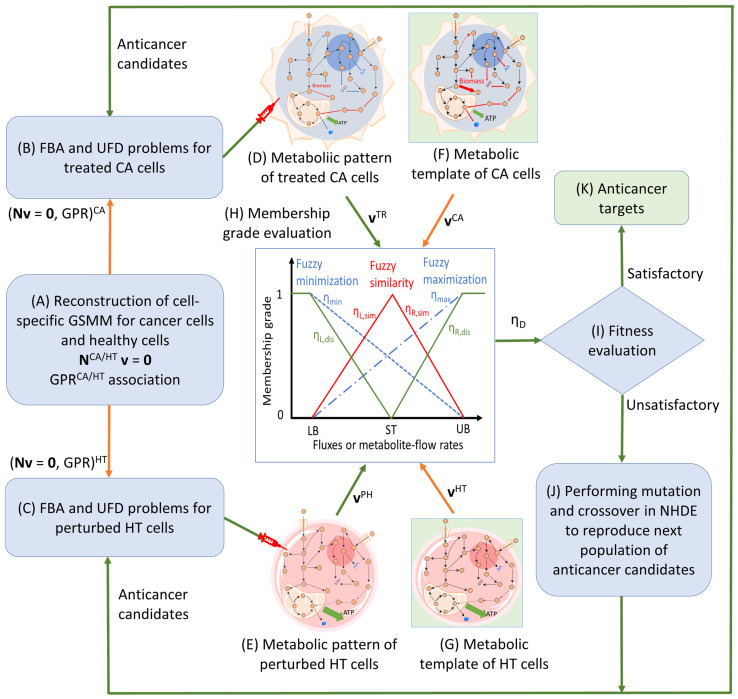
Framework for identifying the anticancer target discovery platform. (**A**) Reconstruction of tissue-specific GSMMs and gene–protein–reaction models for CA cells and HT cells. (**B**) Formulation of a parsimonious flux balance analysis problem for treating CA cells. (**C**) Formulation of a parsimonious flux balance analysis problem for perturbed HT cells. (**D**) Obtaining the metabolic pattern of treated CA cells for each anticancer candidate target by conducting the pFBA problem. (**E**) Obtaining the metabolic pattern of perturbed HT cells for each anticancer candidate target by conducting the pFBA problem. (**F**) Deriving the metabolic template of CA cells from clinical data (if available) or computed by conducting the pFBA problem without considering anticancer target regulation. (**G**) Deriving the metabolic template of HT cells from clinical data (if available) or computed by conducting the pFBA problem without considering anticancer target regulation. (**H**) Use of fuzzy set theory to convert fuzzy multiobjective functions into a fuzzy decision (*η_D_*) in a maximizing decision-making problem. (**I**) Evaluation of the fitnesses of all anticancer candidates to make a decision. (**J**) Generating the subsequent set of anticancer candidates by using a nested hybrid differential evolution algorithm if the decision criterion is unsatisfied and then repeating Steps (**B**,**J**) until the maximum iteration assigned by the user is achieved. (**K**) The optimal targets are obtained if the fitness is satisfactory.

In this study, we established an anticancer target discovery (ACTD) framework aimed at identifying potential anticancer targets, as depicted in Figure 6. This framework bears a resemblance to the antiviral target discovery platform. Tissue-specific genome-scale metabolic models (GSMMs) were reconstructed for HCC cells (referred to as CA cells) and their healthy counterparts (referred to as HT cells), as demonstrated in Steps A–D of Figure 5. Subsequently, both models were employed to formulate the pFBA problem, expressed in Equation (1), to assess flux distributions resulting from target treatment and the ensuing perturbations.

Conventional screening strategies apply the death of cancer cells as an evaluation criterion to discover targets. The ACTD framework not only reduces the growth rate of cancer cells but also minimizes the metabolic deviation of the metabolic patterns (Steps D and E) for treated cancer cells (referred to as TR cells) and perturbed healthy cells (referred to as PH cells) in comparison to their healthy counterparts. Metabolic patterns for TR and PH cells are represented as distributions of fluxes and metabolite flow rates resulting from target modulation. The metabolic templates (Steps F and G) of the CA and HT cells are defined as standard levels of fluxes and metabolite flow rates in their corresponding metabolic networks. Although these templates are typically obtained from clinical data, such a genome-scale standard is not currently available. In this study, a pFBA problem was formulated to compute the templates for CA and HT cells, respectively. The metabolic patterns were computed through Steps B and C for each candidate target and then compared against the metabolic templates to identify optimal targets. Subsequently, the metabolic patterns and templates were used to assess multi-objective fuzzy membership functions (Step H) and then transformed into a decision-making problem to maximize decision fitness (η_D_). The fitness value for each anticancer candidate was used as a basis to identify the candidates that achieved a satisfactory grade (Step I). If the decision criterion was not satisfied, a subsequent set of anticancer candidates was generated using a nested hybrid differential evolution algorithm (Step J). The anticancer target was considered optimal if it had a good fit (Step K).

### 3.3. Fuzzy Multi-Objective Hierarchical Optimization Problem

**Figure 7:**
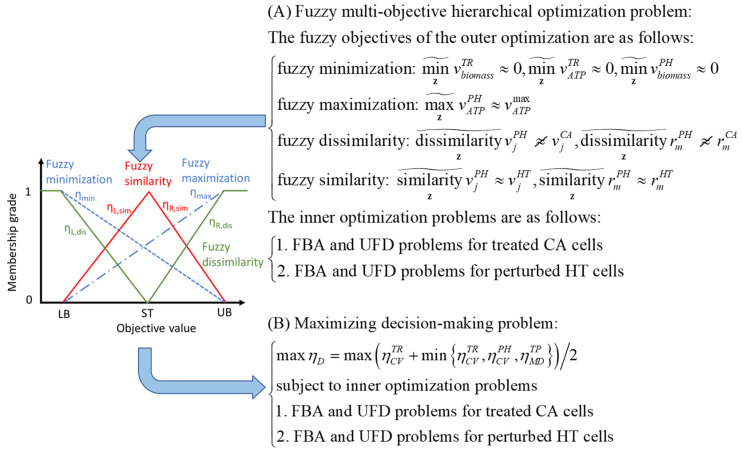
Transformation of a fuzzy multi-objective hierarchical optimization problem into a maximizing decision-making problem using fuzzy membership functions. The lower bound (LB), upper bound (UB), and standard value (ST) are provided by a user. These values can be obtained from clinical data (if available) or estimated from CA and HT templates. One-sided linear membership functions are used to evaluate the fuzzy minimization and maximization (dashed and dot-dashed lines, respectively). Two-sided linear membership functions are used to evaluate the fuzzy similarity and fuzzy dissimilarity (red and green lines, respectively).

The ACTD framework was formulated as a fuzzy multi-objective hierarchical optimization (FMHO) problem, as illustrated in Figure 7A, encompassing four fuzzy objectives. We defined a cell viability grade ($\eta_{CV}^{TR}$) for TR cells as a criterion for evaluating cell mortality in cancer cells after treatment using a target. The cell viability grade for TR cells was determined through the fuzzy minimization of the biomass growth rate and ATP production rate. In contrast, a cell viability grade ($\eta_{CV}^{PH}$) for PH cells was defined as a metric to evaluate the fuzzy minimization of the biomass growth rate and maximization of the ATP synthesis rate for PH cells due to treatment. More than 5000 fluxes and metabolite flow rates were acquired from the corresponding pFBA problems involving TR and PH cells. These values, denoted as metabolic patterns, were then contrasted with their templates to assess the disparities in metabolic distributions between the two models. We incorporated a novel concept of fuzzy dissimilarity to assess differences in the metabolic patterns of the TR and PH cells relative to the metabolic template of the CA cells. Additionally, the degree of similarity between the metabolic patterns of the TR and PH cells and the metabolic template of the HT cells was evaluated by employing fuzzy similarity. Fuzzy dissimilarity and similarity were determined using two-sided membership functions, which yielded a metabolic deviation grade ($\eta_{MD}^{TP}$) that can be used as a metric for evaluating the side effects of treatment. The procedures for computation are detailed in the literature [24] and Supplementary File S6.

### 3.4. Computational Procedures

Based on fuzzy set theory, the FMHO problem illustrated in Figure 7A can be transformed into a maximizing decision-making (MDM) problem, as depicted in Figure 7B. The MDM problem can be solved using the nested hybrid differential evolution (NHDE) algorithm [38]. The optimality conditions of this transformation were proven in a previous study [38]. According to the optimality conditions, a Pareto solution to the FMHO problem can be derived based on the transformed MDM problem. MDM problems are challenging optimization problems that cannot be solved directly using commercially available software [42,43]. The high-dimensional, bilevel, and mixed-integer linear characteristics of these problems make them NP-hard.

**Figure 8:**
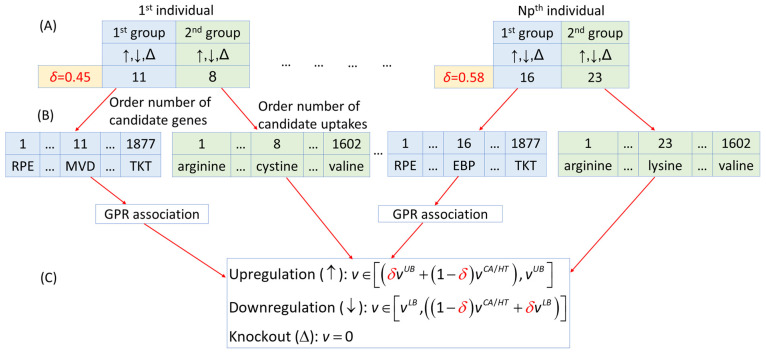
Representation of target individuals comprising candidate genes and nutrient uptakes. (**A**) Each two-group target is represented by a structure array that comprises a combination of order numbers for each two-group target, a regulated strength parameter (*δ*), and its corresponding regulation (up, down, or knockout). (**B**) The order numbers are mapped to the corresponding genes and nutrient uptakes. (**C**) The candidate genes are used to restrict the lower and upper bounds of the fluxes through the GPR association, and the candidate uptakes are also limited by their lower and upper bounds.

In this study, we applied the NHDE algorithm, which is a stochastic and parallel direct search algorithm based on procedures in hybrid differential evolution (HDE) [44], an extension of the original differential evolution algorithm [45]. The computational procedures of the NHDE algorithm are detailed in Table 4 and Supplementary File S6. The initialization process involved randomly generating a population of *Np* individuals (***z****_i_*) to cover the entire search space uniformly. A structure array was used to represent the order number of a candidate target, its corresponding regulation, and the regulated strength parameter. Additionally, a two-group strategy was established to represent candidate targets and identify combinations of candidate genes and nutrient uptakes, as illustrated in Figure 8.

## 4. Conclusions

HCC, the predominant subtype of primary liver cancer, is a leading cause of cancer-related mortality globally. Metabolic dysregulation is increasingly recognized as a significant risk factor for HCC, playing a crucial role in tumorigenesis and progression. Similar to other tumors, HCC tumors are marked by the neoplastic transformation of normal hepatocytes through substantial metabolic rewiring. This paper introduces an ACTD framework to identify potential biomarkers and anticancer targets for HCC treatment. The framework involves reconstructing tissue-specific GSMMs for both cancer cells and their healthy counterparts and employing them to formulate parsimonious flux variability analysis problems for biomarker identification. Furthermore, this study employed an FMHO framework to identify promising anticancer targets for HCC treatment.

Quartile weighting factors were employed in the pFBA problems for both models instead of equal weightings to calculate the flux patterns of the CA and HT models. Biomarkers are commonly identified using both flux patterns to evaluate log2 fold change and a *p*-value less than 0.05. This approach makes it difficult to discern the overlapping region of the distributions. We used confidence limits to categorize the flux patterns into seven groups and then to identify completely decreased or increased biomarkers. According to this classification, two secretions and thirteen uptakes were identified as potential biomarkers. Among these uptakes, eight were essential amino acids and two were conditional essential amino acids, indicating higher amino acid uptake rates in cancer cells relative to healthy cells.

The nutrient composition can significantly influence target identification. Within the ACTD framework, we employed ten different media to explore this effect and identify corresponding anticancer enzymes. The findings indicated that the identifiability of an enzyme participating in the cholesterol biosynthetic pathway may vary when it is used in conjunction with a cholesterol uptake medium. Computational results indicated that enzymes in the cholesterol biosynthetic pathway can be identified when a cholesterol uptake reaction does not occur in the medium used. Moreover, enzymes in the cholesterol biosynthetic pathway were identifiable in a cholesterol-free cell culture medium. A two-group representation was also introduced in the ACTD framework to identify target combinations, which involved pairing a single-target enzyme with an additional exchange reaction. Computational results showed that cell viability grades for all two-target combinations were higher than those for their corresponding one-target enzymes, suggesting that combining a target enzyme with the modulation of an additional exchange reaction can effectively reduce the biomass growth rate and ATP production rate in treated cancer cells. Additionally, most two-target combinations exhibited an enhanced metabolic deviation grade relative to their corresponding single-target enzymes.

Metabolic deviation grades assess variations in metabolic patterns between perturbed healthy cells and their corresponding cancerous and healthy templates, facilitating the prediction of potential side effects associated with the identified targets. The CA and HT templates serve as standard benchmarks for evaluating metabolic deviation grades. In this study, the pFBA problem was applied to calculate the flux distributions of the CA and HT cells, utilized as the standard templates. In future studies, the integration of genome-scale clinical data, where available, could refine these templates, potentially enhancing side effect prediction and the relevance of the approach.

## Figures and Tables

**Table 1:** Cell viability (CV) grades and metabolic deviation (MD) grades of identified enzymes in various nutrient media. D/T was obtained from the DepMap portal (https://depmap.org/portal/, accessed on 21 August 2023) and is defined as the ratio of the cell death number (N) and the total number (T) of colon cancer cell lines used in the experimental test. “No. Drugs” denotes the number of drugs retrieved from DrugBank (https://go.drugbank.com/, accessed on 21 August 2023) that modulate each enzyme. “-- ” indicates that the enzyme was unidentifiable by the nutrient medium used. “*” indicates that CTSA is the representative of the duplicate enzymes (CTSA, GLB1, NEU1, and PSAP).

	DMEM		HAM		HPLM		RPMI		VMH		D/T	No. Drugs	Pathway
Target	CV	MD	CV	MD	CV	MD	CV	MD	CV	MD			
CMPK1	0.869	0.472	0.842	0.594	--	--	--	--	--	--	40/43	9	Gemcitabine pathway
DCTD	0.869	0.451	0.842	0.561	0.980	0.479	--	--	--	--	1/43	1	Gemcitabine pathway
PGS1	0.833	0.431	0.842	0.625	0.980	0.518	0.980	0.496	0.980	0.530	36/43	0	Glycerophospholipid biosynthesis
CRLS1	0.833	0.424	0.842	0.586	0.980	0.479	0.980	0.550	0.980	0.565	25/43	0	Glycerophospholipid biosynthesis
MVD	0.833	0.421	--	--	0.980	0.500	0.980	0.551	--	--	31/43	0	Cholesterol biosynthesis
MVK	0.833	0.421	--	--	0.980	0.510	0.980	0.507	--	--	34/43	1	Cholesterol biosynthesis
PMVK	0.833	0.421	--	--	0.980	0.500	0.980	0.507	--	--	10/43	0	Cholesterol biosynthesis
SC5D	0.833	0.411	--	--	0.980	0.511	0.980	0.559	--	--	0/43	0	Cholesterol biosynthesis
HMGCR	0.833	0.411	--	--	0.980	0.519	0.980	0.545	--	--	43/43	20	Cholesterol biosynthesis
ADSL	0.833	0.410	0.842	0.615	0.980	0.506	0.980	0.516	--	--	28/43	0	Metabolism of nucleotides
ADSS2	0.833	0.408	0.842	0.604	0.980	0.506	0.980	0.506	--	--	22/43	2	Metabolism of nucleotides
LSS	0.833	0.407	--	--	0.980	0.489	0.980	0.567	--	--	0/43	2	Cholesterol biosynthesis
SQLE	0.833	0.407	--	--	0.980	0.541	0.980	0.567	--	--	1/43	4	Cholesterol biosynthesis
GK	0.833	0.406	0.842	0.562	0.980	0.482	--	--	--	--	0/43	0	Glycerophospholipid biosynthesis
DHCR24	0.833	0.405	--	--	0.980	0.479	0.980	0.520	--	--	0/43	0	Cholesterol biosynthesis
FDFT1	0.833	0.402	--	--	0.980	0.507	0.980	0.530	--	--	2/43	1	Cholesterol biosynthesis
EBP	0.833	0.402	--	--	0.980	0.510	0.980	0.507	--	--	0/43	0	Cholesterol biosynthesis
CTSA *	0.812	0.479	--	--	--	--	0.980	0.565	0.980	0.448	0/43	3	Sphingolipid metabolism
GMPR2	0.534	0.371	--	--	0.447	0.465	0.564	0.375	--	--	0/43	1	Metabolism of nucleotides
CEPT1	0.307	0.288	0.308	0.328	--	--	--	--	--	--	9/43	2	Glycerophospholipid biosynthesis
PTDSS2	0.234	0.317	0.250	0.379	--	--	--	--	0.564	0.320	0/43	1	Glycerophospholipid biosynthesis
NSDHL	0.224	0.269	0.842	0.594	0.273	0.279	0.265	0.290			0/43	1	Cholesterol biosynthesis

**Table 2:** Cell viability (CV) grades and metabolic deviation (MD) grades of identified enzymes in various nutrient media with or without cholesterol uptake. “--” indicates that the enzyme was unidentifiable by the nutrient medium used. “*” indicates that CTSA is the representative of duplicate enzymes (CTSA, GLB1, NEU1, and PSAP).

	DMEM+Cholesterol		HAM−Cholesterol		HPLM+Cholesterol		RPMI+Cholesterol		VMH−Cholesterol	
Target	CV	MD	CV	MD	CV	MD	CV	MD	CV	MD
CMPK1	0.869	0.480	0.842	0.594	--	--	--	--	--	--
DCTD	0.869	0.449	0.842	0.560	0.980	0.477	--	--	--	--
PGS1	0.833	0.421	0.842	0.590	0.980	0.563	0.980	0.457	0.980	0.550
CRLS1	0.833	0.423	0.842	0.579	0.980	0.486	0.980	0.510	0.980	0.547
MVD	--	--	0.842	0.656	--	--	--	--	0.980	0.574
MVK	--	--	0.842	0.656	--	--	--	--	0.980	0.574
PMVK	--	--	0.842	0.656	--	--	--	--	0.980	0.574
SC5D	--	--	0.842	0.617	--	--	--	--	0.980	0.527
HMGCR	--	--	0.842	0.600	--	--	--	--	0.980	0.533
ADSL	0.833	0.413	0.842	0.604	0.980	0.480	0.980	0.454	--	--
ADSS2	0.833	0.414	0.842	0.596	0.980	0.499	0.980	0.476	--	--
LSS	--	--	0.842	0.601	--	--	--	--	0.980	0.574
SQLE	--	--	0.842	0.601	--	--	--	--	0.980	0.574
GK	0.833	0.438	0.842	0.567	0.980	0.501	--	--	--	--
DHCR24	--	--	0.842	0.590	--	--	--	--	0.980	0.555
FDFT1	--	--	0.842	0.596	--	--	--	--	0.980	0.580
EBP	--	--	0.842	0.604	--	--	--	--	0.980	0.567
CTSA *	0.812	0.486	--	--	--	--	0.980	0.533	0.980	0.460
GMPR2	--	--	--	--	0.447	0.428	0.564	0.373	--	--
CEPT1	0.307	0.292	0.308	0.321	--	--	--	--	--	--
PTDSS2	--	--	--	--	--	--	--	--	0.564	0.324
NSDHL	--	--	--	--	--	--	--	--	0.273	0.275

**Table 3:** Combinations of various single-target enzymes with additional exchange reactions in a nutrient medium. “↓” indicates that an enzyme is downregulated or an exchange reaction is to be taken up. “Δ” denotes that an enzyme is knocked out or an exchange reaction is to be blocked. “↑” indicates that an enzyme is upregulated or an exchange reaction is to be secreted.

DMEM			HAM			VMH		
Two-Target Combinations	CV	MD	Two-Target Combinations	CV	MD	Two-Target Combinations	CV	MD
(CMPK1^Δ^, R_EX_galt[e]^↓^)	0.980	0.432	(CMPK1^Δ^, R_EX_glyc_R[e]^↓^)	0.980	0.526	(CMPK1^Δ^, R_EX_thymd[e]^Δ^)	0.980	0.552
(DCTD^↓^, R_EX_glyc2p[e]^↓^)	0.980	0.475	(DCTD^↓^, R_EX_glyc_R[e]^↓^)	0.980	0.493	(DCTD^Δ^, R_EX_trp_L[e]^Δ^)	0.980	0.577
(PGS1^Δ^, R_EX_glyc3p[e]^↓^)	0.980	0.494	(PGS1^Δ^, R_EX_CE5304[e]^↓^)	0.980	0.561	(PGS1^Δ^, R_EX_sphs1p[e]^↓^)	0.980	0.582
(CRLS1^Δ^, R_EX_glyc2p[e]^↓^)	0.980	0.477	(CRLS1^Δ^, R_EX_CE5304[e]^↓^)	0.980	0.564	(CRLS1^Δ^, R_EX_lstnm1[e]^Δ^)	0.980	0.577
(MVD^Δ^, R_EX_icit[e]^↓^)	0.980	0.476	(MVD^Δ^, R_EX_chsterol[e]^Δ^)	0.842	0.656	(MVD^Δ^, R_EX_thymd[e]^Δ^)	0.980	0.533
(MVK^Δ^, R_EX_34dhpha[e]^↓^)	0.980	0.464	(MVK^Δ^, R_EX_thymd[e]^Δ^)	0.842	0.610	(MVK^↓^, R_EX_asn_L[e]^Δ^)	0.980	0.370
(PMVK^↓^, R_EX_glyc_R[e]^↑^)	0.980	0.455	(PMVK^↓^, R_EX_phe_L[e]^Δ^)	0.842	0.518	(PMVK^Δ^, R_EX_chsterol[e]^Δ^)	0.980	0.574
(SC5D^Δ^, R_EX_icit[e]^↓^)	0.980	0.508	(SC5D^Δ^, R_EX_thymd[e]^Δ^)	0.842	0.603	(SC5D^Δ^, R_EX_chsterol[e]^Δ^)	0.980	0.527
(HMGCR^Δ^, R_EX_glyc[e]^↓^)	0.980	0.446	(HMGCR^Δ^, R_EX_met_L[e]^Δ^)	0.842	0.603	(HMGCR^Δ^, R_EX_phe_L[e]^Δ^)	0.980	0.399
(LSS^Δ^, R_EX_icit[e]^↓^)	0.980	0.517	(LSS^Δ^, R_EX_his_L[e]^Δ^)	0.842	0.611	(LSS^Δ^, R_EX_chsterol[e]^Δ^)	0.980	0.574
(SQLE^Δ^, R_EX_icit[e]^↓^)	0.980	0.524	(SQLE^Δ^, R_EX_asn_L[e]^Δ^)	0.842	0.512	(SQLE^↓^, R_EX_trp_L[e]^Δ^)	0.980	0.574
(DHCR24^Δ^, R_EX_34dhpha[e]^↓^)	0.980	0.474	(DHCR24^Δ^, R_EX_his_L[e]^Δ^)	0.842	0.593	(DHCR24^Δ^, R_EX_chsterol[e]^Δ^)	0.980	0.555
(CTPS1^Δ^, R_EX_icit[e]^↓^)	0.980	0.481	(CTPS1^Δ^, R_EX_strch1[e]^↓^)	0.959	0.555	(CTPS1^↓^, R_EX_thymd[e]^Δ^)	0.980	0.552
(ATIC^Δ^, R_EX_acald[e]^↑^)	0.944	0.341	(ATIC^Δ^, R_EX_lnlc[e]^Δ^)	0.842	0.593	(ATIC^↓^, R_EX_thymd[e]^Δ^)	0.980	0.562
(CDIPT^Δ^, R_EX_acald[e]^↑^)	0.941	0.345	(CDIPT^Δ^, R_EX_trp_L[e]^Δ^)	0.842	0.602	(CDIPT^Δ^, R_EX_thymd[e]^Δ^)	0.980	0.553
(PRPS1L1^Δ^, R_EX_HC01444[e]^↑^)	0.911	0.424	(PRPS1L1^Δ^, R_EX_met_L[e]^Δ^)	0.842	0.617	(PRPS1L1^Δ^, R_EX_trp_L[e]^Δ^)	0.980	0.549
(GLA^↓^, R_EX_thymd[e]^Δ^)	0.896	0.433	(GLA^↓^, R_EX_thymd[e]^Δ^)	0.842	0.609	(GLA^↓^, R_EX_mi1p_D[e]^Δ^)	0.980	0.547
(SLC2A13^↓^, R_EX_thymd[e]^Δ^)	0.884	0.440	(SLC2A13^↓^, R_EX_thymd[e]^Δ^)	0.842	0.613	(SLC2A13^↓^, R_EX_trp_L[e]^Δ^)	0.980	0.558
(RPE^Δ^, R_EX_trp_L[e]^Δ^)	0.869	0.425	(RPE^Δ^, R_EX_met_L[e]^Δ^)	0.842	0.455	(RPE^Δ^, R_EX_mi1p_D[e]^Δ^)	0.980	0.555
(NSDHL^Δ^, R_EX_chsterol[e]^↓^, R_EX_ga1_hs[e]^Δ^)	0.812	0.489	(NSDHL^Δ^, R_EX_met_L[e]^Δ^)	0.842	0.616	(NSDHL^↓^, R_EX_mi1p_D[e]^Δ^)	0.980	0.528
(MVD^Δ^, GOT1^Δ^)	0.980	0.491	(MVD^Δ^, CRLS1^Δ^)	0.842	0.592	(MVD^↓^, PSAP^Δ^)	0.980	0.434
(MVK^Δ^, GMPR2^↓^)	0.980	0.503	(MVK^↓^, ADSS2^Δ^)	0.842	0.627	(MVK^Δ^, PGS1^Δ^)	0.980	0.567
(PMVK^Δ^, GLA^↓^)	0.980	0.479	(PMVK^Δ^, ADSL^↓^)	0.842	0.605	(PMVK^↓^, CRLS1^Δ^)	0.980	0.507
(SC5D^Δ^, SLC25A10^Δ^)	0.980	0.454	(SC5D^↓^, CMPK1^Δ^)	0.842	0.599	(SC5D^↓^, PSAP^Δ^)	0.980	0.446
(HMGCR^Δ^, MPC2^Δ^)	0.980	0.480	(HMGCR^Δ^, NEU3^Δ^)	0.710	0.604	(HMGCR^Δ^, SLC27A4^Δ^)	0.980	0.495
(LSS^Δ^, GLA^↓^)	0.980	0.491	(LSS^Δ^, CMPK1^Δ^)	0.842	0.604	(LSS^↓^, PSAP^Δ^)	0.980	0.459
(SQLE^Δ^, SLC17A1^↓^)	0.941	0.487	(SQLE^↓^, PGS1^Δ^)	0.842	0.581	(SQLE^↓^, CRLS1^↓^)	0.980	0.547
(DHCR24^Δ^, MPC2^Δ^)	0.980	0.480	(DHCR24^Δ^, ADSS2^Δ^)	0.842	0.587	(DHCR24^Δ^, SLC27A4^Δ^)	0.980	0.506
(FDFT1^Δ^, GLA^↓^)	0.980	0.533	(FDFT1^Δ^, CMPK1^Δ^)	0.842	0.609	(FDFT1^Δ^, PGS1^Δ^)	0.980	0.552
(EBP^Δ^, GLA^↓^)	0.980	0.507	(EBP^Δ^, PGS1^↓^)	0.842	0.609	(EBP^Δ^, CRLS1^Δ^)	0.980	0.515
(NSDHL^↓^, CRLS1^Δ^)	0.834	0.392	(NSDHL^↓^, ADSS2^Δ^)	0.842	0.600	(NSDHL^Δ^, CRLS1^Δ^)	0.980	0.525

**Table 4:** Computational procedures of the nested hybrid differential evolution algorithm to solve the maximizing decision-making problem.

NHDE
Represent and initialize for a population of target individuals $(z^{0}{)}_{i}=\mathrm{uniformInt}\left(z^{\min},\;z^{\max}\right),\;i=1,\ldots ,\;N_{p}$ Each individual is represented as a structure array generated by a mixed-integer random array between **z**^min^ and **z**^max^ with a uniform distribution Use mutation with a rounding operation to generate a new population of target individuals $({\hat{z}}^{G}{)}_{i}=\mathrm{INT}\left\{(z^{G}{)}_{p}+\right.\left.\rho^{G}\left[(z^{G}{)}_{j}-(z^{G}{)}_{k}+(z^{G}{)}_{l}-(z^{G}{)}_{m}\right]\right\}$ Use crossover operation to reproduce the next target individuals $z_{ji}^{G}=\left\{\begin{array}{l} z_{ji}^{G-1},\mathrm{if}\;a\;\mathrm{random}\;\mathrm{number}>C_{R}\\ {\hat{z}}_{ji}^{G},\mathrm{otherwise},j=1,\ldots ,n;\;i=1,\ldots ,N_{p} \end{array}\right.$ Use restriction operation to restrain a individual within its bounds $z_{ji}^{G}=\left\{\begin{array}{l} z_{ji}^{G-1},\;\mathrm{if}\;a\;\mathrm{random}\;\mathrm{number}>C_{R}\;\;\;\;\;\;\\ {\hat{z}}_{ji}^{G},\;\mathrm{otherwise},\;j=1,\ldots ,n;i=1,\ldots ,N_{p} \end{array}\right.$ Use selection and evaluation to determine the surviving individuals at the next generation (a) For each target individual, solve the inner pFBA problem for the treated CA model and perturbed HT model, respectively. (b) Compute the fitness for each target individual (**z***_i_*) of the maximizing decision-making problem fitnes $s_{z_{i}}={\left(\eta_{D}\right)}_{z_{i}}-\Psi_{z_{i}}$ where $\Psi_{z_{i}}$ is a positive penalty to discipline the target individual (**z***_i_*) if its corresponding solution is infeasible. (c) Use one-to-one competition between the parent individual and its corresponding offspring, and select the best individual from the population of individuals. Use migration operation to prevent clustering around the best individual in a subsequent iteration, achieving a premature solution. $\left(z^{G}{)}_{i}=\mathrm{uniformInt}(z^{min},z^{max}),\;\mathrm{if}\;\zeta \leq \varepsilon =[0,1\right]$ Repeat steps 2 to 6

## Data Availability

The source programs of the anticancer target discovery platform and the cell-specific genome-scale metabolic models are coded by the General Algebraic Modeling System (GAMS, https://www.gams.com/, accessed on 16 July 2022) and are available at http://doi.org/10.5281/zenodo.10771499. The data of this study are available in the TCGA database (https://www.cancer.gov/about-nci/organization/ccg/research/structural-genomics/tcga, accessed on 10 April 2023) and the Virtual Metabolic Human (https://www.vmh.Life, accessed on 10 April 2023).

## Supplementary Materials

The following supporting information can be downloaded at: https://www.mdpi.com/article/10.3390/molecules29112594/s1, Supplementary File S1. A list of CA and HT models and uptake reactions in different nutrient media; Supplementary File S2. A compilation of one-target enzymes identified through the ACTD framework; Supplementary File S3. A compilation of combinations of two targets identified through the ACTD framework; Supplementary File S4. A list of differential expressions of exchange reactions; Supplementary File S5. A list of differential expressions of metabolite flow rates; Supplementary File S6. Computational procedures of the nested hybrid differential evolution algorithm. References [38,44,45] are cited in the supplementary materials.

## References

[1] Zhou Z., Xu M.J., Gao B. (2016). Hepatocytes: A key cell type for innate immunity. Cell. Mol. Immunol..

[2] Tenen D.G., Chai L., Tan J.L. (2021). Metabolic alterations and vulnerabilities in hepatocellular carcinoma. Gastroenterol. Rep..

[3] Siegel R.L., Miller K.D., Wagle N.S., Jemal A. (2023). Cancer statistics, 2023. CA Cancer J. Clin..

[4] Sung H., Ferlay J., Siegel R.L., Laversanne M., Soerjomataram I., Jemal A., Bray F. (2021). Global cancer statistics 2020: GLOBOCAN estimates of incidence and mortality worldwide for 36 cancers in 185 countries. CA Cancer J. Clin..

[5] Satriano L., Lewinska M., Rodrigues P.M., Banales J.M., Andersen J.B. (2019). Metabolic rearrangements in primary liver cancers: Cause and consequences. Nat. Rev. Gastroenterol. Hepatol..

[6] Pavlova N.N., Thompson C.B. (2016). The emerging hallmarks of cancer metabolism. Cell Metab..

[7] DeBerardinis R.J., Chandel N.S. (2016). Fundamentals of cancer metabolism. Sci. Adv..

[8] Pavlova N.N., Zhu J., Thompson C.B. (2022). The hallmarks of cancer metabolism: Still emerging. Cell Metab..

[9] Konda P., Garinet SVan Allen EMViswanathan S.R. (2023). Genome-guided discovery of cancer therapeutic targets. Cell Rep..

[10] Finley L.W.S. (2023). What is cancer metabolism?. Cell.

[11] Ge M., Luo J., Wu Y., Shen G., Kuang X. (2024). The biological essence of synthetic lethality: Bringing new opportunities for cancer therapy. Oncology.

[12] Su M.C., Lee Am Zhang W., Maeser D., Gruener R.F., Deng Y., Huang R.S. (2024). Computational modeling to identify drugs targeting metastatic castration-resistant prostate cancer characterized by heightened glycolysis. Pharmaceuticals.

[13] Folger O., Jerby L., Frezza C., Gottlieb E., Ruppin E., Shlomi T. (2011). Predicting selective drug targets in cancer through metabolic networks. Mol. Syst. Biol..

[14] Yizhak K., Gaude E., Le Dévédec S., Waldman Y.Y., Stein G.Y., Water B.V.D., Frezza C., Ruppin E. (2014). Phenotype-based cell-specific metabolic modeling reveals metabolic liabilities of cancer. eLife.

[15] Ghaffari P., Mardinoglu A., Nielsen J. (2015). Cancer metabolism: A modeling perspective. Front. Physiol..

[16] Jerby L., Ruppin E. (2012). Predicting drug targets and biomarkers of cancer via genome-scale metabolic modeling. Clin. Cancer Res..

[17] Lewis N.E., Abdel-Haleem A.M. (2013). The evolution of genome-scale models of cancer metabolism. Front. Physiol..

[18] Nilsson A., Nielsen J. (2017). Genome scale metabolic modeling of cancer. Metab. Eng..

[19] Robinson J.L., Nielsen J. (2017). Anticancer drug discovery through genome-scale metabolic modeling. Curr. Opin. Syst. Biol..

[20] Wu H.Q., Cheng M.L., Lai J.M., Wu H.H., Chen M.C., Liu W.H., Wu W.H., Chang P.M.H., Huang C.Y.F., Tsou A.P. (2017). Flux balance analysis predicts Warburg-like effects of mouse hepatocyte deficient in miR-122a. PLoS Comput. Biol..

[21] Wang F.S., Wu W.H., Hsiu W.S., Liu Y.J., Chuang K.W. (2020). Genome-scale metabolic modeling with protein expressions of normal and cancerous colorectal tissues for oncogene inference. Metabolites.

[22] Wang Y.T., Lin M.R., Chen W.C., Wu W.H., Wang F.S. (2021). Optimization of a modeling platform to predict oncogenes from genome-scale metabolic networks of non-small-cell lung cancers. FEBS OpenBio.

[23] Cheng C.T., Wang T.Y., Chen P.R., Wu W.H., Lai J.M., Chang P.M.H., Hong Y.R., Huang C.Y.F., Wang F.S. (2021). Computer-aided design for identifying anticancer targets in genome-scale metabolic models of colon cancer. Biology.

[24] Wang F.S., Chen P.R., Chen T.Y., Zhang H.X. (2022). Fuzzy optimization for identifying anticancer targets with few side effects in constraint-based models of head and neck cancer. R. Soc. Open Sci..

[25] MohammadiPeyhani H., Chiappino-Pepe A., Haddadi K., Hafner J., Hadadi N., Hatzimanikatis V. (2021). NICEdru, a workflow for rational drug design and systems-level analysis of drug metabolism. eLife.

[26] You Y., Lai X., Pan Y., Zheng H., Vera J., Liu S., Deng S., Zhang L. (2022). Artificial intelligence in cancer target identification and drug discovery. Signal Transduct. Target. Ther..

[27] Cui W., Aouidate A., Wang S., Yu Q., Li Y., Yuan S. (2020). Discovering anti-cancer drugs via computational methods. Front. Pharmacol..

[28] Huang A., Garraway L.A., Ashworth A., Weber B. (2019). Synthetic lethality as an engine for cancer drug target discovery. Nat. Rev. Drug Discov..

[29] Rinschen M.M., Ivanisevic J., Giera M., Siuzdak G. (2019). Identification of bioactive metabolites using activity metabolomics. Nat. Rev. Mol. Cell Biol..

[30] Lewis J.E., Kemp M.L. (2021). Integration of machine learning and genome-scale metabolic modeling identifies multi-omics biomarkers for radiation resistance. Nat. Commun..

[31] Moškon M., Režen T. (2023). Context-specific genome-scale metabolic modelling and its application to the analysis of COVID-19 metabolic signatures. Metabolites.

[32] Jenior M.L., Moutinho T.J., Dougherty B.V., Papin J.A. (2020). Transcriptome-guided parsimonious lux analysis improves predictions with metabolic Networks in complex environments. PLoS Comput. Biol..

[33] National Cancer Institute of U.S. The Cancer Genome Atlas Program. Department of Health and Human Services. https://www.cancer.gov/about-nci/organization/ccg/research/structural-genomics/tcga.

[34] Schultz A., Qutub A.A. (2016). Reconstruction of tissue-specific metabolic networks using CORDA. PLoS Comput. Biol..

[35] Heiden M.G.V., Locasale J.W., Swanson K.D., Sharfi H., Heffron G.J., Daniel Amador-Noguez D., Christofk H.R., Wagner G., Rabinowitz J.D., Asara J.M. (2010). Evidence for an alternative glycolytic pathway in rapidly proliferating cells. Science.

[36] Jain M., Nilsson R., Sharma S., Madhusudhan N., Kitami T., Souza A.L., Kafri R., Kirschner M.W., Clish C.B., Mootha V.K. (2012). Metabolite profiling identifies a key role for glycine in rapid cancer cell proliferation. Science.

[37] Asgari Y., Zabihinpour Z., Salehzadeh-Yazdi A., Schreiber F., Masoudi-Nejad A. (2015). Alterations in cancer cell metabolism: The Warburg effect and metabolic adaptation. Genomics.

[38] Wang F.S., Wang T.Y., Wu W.H. (2022). Fuzzy multiobjective hierarchical optimization with application to identify antienzymes of colon cancer cells. J. Taiwan Inst. Chem. Eng..

[39] Wishart D.S., Feunang Y.D., Guo A.C., Lo E.J., Marcu A., Grant J.R., Sajed T., Johnson D., Li C., Sayeeda Z. (2018). DrugBank 5.0: A major update to the DrugBank database for 2018. Nucleic Acids Res..

[40] Brunk E., Sahoo S., Zielinski D.C., Altunkaya A., Dräger A., Mih N., Gatto F., Nilsson A., Andres Gonzalez A.P., Aurich M.K. (2018). Recon3D enables a three-dimensional view of gene variation in human metabolism. Nat. Biotechnol..

[41] Zur H., Ruppin E., Shlomi T. (2010). iMAT: An integrative metabolic analysis tool. Bioinformatics.

[42] Vaskan P., Guillén-Gosálbez G., Sorribas A., Alves R., Jiménez L. (2014). Multi-level optimization framework applied to the systematic evaluation of metabolic objective functions. Comput. Aided Chem. Eng..

[43] Pozo C., Miró A., Guillén-Gosálbez G., Sorribas A., Alves R., Jiménez L. (2015). Global optimization of hybrid kinetic/FBA models via outer-approximation. Comput. Chem. Eng..

[44] Chiou J.P., Wang F.S. (1999). Hybrid method of evolutionary algorithms for static and dynamic optimization problems with application to a fed-batch fermentation process. Comput. Chem. Eng..

[45] Storn R., Price K. (1997). Differential evolution—A simple and efficient heuristic for global optimization over continuous spaces. J. Glob. Optim..

