# Supplementary material for: Identification of Anticancer Enzymes and Biomarkers for Hepatocellular Carcinoma through Constraint-Based Modeling
Source: Molecules. 2024 May 31;29(11):2594. doi: 10.3390/molecules29112594 (PMC11173608; doi:10.3390/molecules29112594)
Supplement: Supplementary file 1 [file molecules-29-02594-s001.zip › Supplementary File S6-ComputationalProcedures.pdf]

Additional File 1:

Computational procedures of ACTD framework and the NHDE algorithm for solving the derived optimization problem.

Three figures and one table are involved in the Supplementary File S1 to describe the methodologies and computational procedures of the anticancer target discovery (ACTD) platform. The figure and table legends are listed as follows:

Figure S1. Computational procedures to obtain the optimal fluxes and metabolite flow rates provided as CA and HT templates.

Figure S2. Evaluation of membership grades for fuzzy minimization, fuzzy maximization, fuzzy similarity and fuzzy dissimilarity.

Figure S3. Flowchart of the parallel search algorithm in NHDE

Table S1. Basic operations for the original DE and NHDE algorithms

The ACTD platform is to formulate as a fuzzy multi-objective hierarchical optimization problem as follows:

Fuzzy multi-objective hierarchical optimization problem:

The fuzzy objectives of the outer optimization are as follows:

$$\begin{cases} \text{fuzzy minimization: } \widetilde{\min_{\mathbf{z}}}\, v_{biomass}^{TR} \approx 0, \widetilde{\min_{\mathbf{z}}}\, v_{ATP}^{TR} \approx 0, \widetilde{\min_{\mathbf{z}}}\, v_{biomass}^{PH} \approx 0 \\ \text{fuzzy maximization: } \widetilde{\max_{\mathbf{z}}}\, v_{ATP}^{PH} \approx v_{ATP}^{\max} \\ \text{fuzzy similarity: } \widetilde{\text{similarity}_{\mathbf{z}}}\, v_j^{TR/PH} \approx v_j^{HT}, \widetilde{\text{similarity}_{\mathbf{z}}}\, r_m^{TR/PH} \approx r_m^{HT} \\ \text{fuzzy dissimilarity: } \widetilde{\text{dissimilarity}_{\mathbf{z}}}\, v_j^{TR/PH} \not\approx v_j^{CA}, \widetilde{\text{dissimilarity}_{\mathbf{z}}}\, r_m^{TR/PH} \not\approx r_m^{CA} \end{cases}$$

The inner optimization problems are as follows:

$$\begin{cases} \text{Treated CA model:} \\ \text{FBA problem:} \qquad\qquad\qquad\qquad \text{UFD problem:} \\ \begin{cases} \max_{\mathbf{v}_{f/b}} v_{biomass} \\ \text{subject to} \\ \quad \mathbf{N}^{CA}\left(\mathbf{v}_f - \mathbf{v}_b\right) = \mathbf{0} \\ \quad v_{f/b,i}^{LB,TR} \le v_{f/b,i} \le v_{f/b,i}^{UB,TR}, i \in \Omega^{TR} \\ \quad v_{f/b,j}^{LB} \le v_{f/b,j} \le v_{f/b,j}^{UB}, j \notin \Omega^{TR} \end{cases} \quad \begin{cases} \min_{\mathbf{v}_{f/b}} \sum_{k \in \Omega^{Int}} c_k^{CA}\left(v_{f,k} + v_{b,k}\right) \\ \text{subject to} \\ \quad \mathbf{N}^{CA}\left(\mathbf{v}_f - \mathbf{v}_b\right) = \mathbf{0} \\ \quad v_{f/b,i}^{LB,TR} \le v_{f/b,i} \le v_{f/b,i}^{UB,TR}, i \in \Omega^{TR} \\ \quad v_{f/b,j}^{LB} \le v_{f/b,j} \le v_{f/b,j}^{UB}, j \notin \Omega^{TR} \\ \quad v_{biomass} \ge v_{biomass}^{*} \end{cases} \\ \text{Perturbed HT model:} \\ \text{FBA problem:} \qquad\qquad\qquad\qquad \text{UFD problem:} \\ \begin{cases} \max_{\mathbf{v}_{f/b}} v_{ATP} \\ \text{subject to} \\ \quad \mathbf{N}^{HT}\left(\mathbf{v}_f - \mathbf{v}_b\right) = \mathbf{0} \\ \quad v_{f/b,i}^{LB,TR} \le v_{f/b,i} \le v_{f/b,i}^{UB,TR}, i \in \Omega^{TR} \\ \quad v_{f/b,j}^{LB} \le v_{f/b,j} \le v_{f/b,j}^{UB}, j \notin \Omega^{TR} \end{cases} \quad \begin{cases} \min_{\mathbf{v}_{f/b}} \sum_{k \in \Omega^{Int}} c_k^{HT}\left(v_{f,k} + v_{b,k}\right) \\ \text{subject to} \\ \quad \mathbf{N}^{HT}\left(\mathbf{v}_f - \mathbf{v}_b\right) = \mathbf{0} \\ \quad v_{f/b,i}^{LB,TR} \le v_{f/b,i} \le v_{f/b,i}^{UB,TR}, i \in \Omega^{TR} \\ \quad v_{f/b,j}^{LB} \le v_{f/b,j} \le v_{f/b,j}^{UB}, j \notin \Omega^{TR} \\ \quad v_{ATP} \ge v_{ATP}^{*} \end{cases} \end{cases} \qquad \text{(S1)}$$

where the stochiometric matrices, $\mathbf{N}^{CA}$ and $\mathbf{N}^{HT}$, for CA and HT models are reconstructed using Step A-D in Figure 1 of the main text. Our previous study [1] used the identical weighting factors, i.e. $c_k^{CA/HT}=1$ for UFD problems. In the present study, the RNA-seq expressions for CA and HT cells are not only used to reconstruct cell-specific GSMMs but also to set the weighting factors c_k^{CA} and c_k^{HT} for UFD problems to obtain uniform flux distributions. The weighting factors depended on quartile

confidence classification using the RNA-seq expression of each cell. The four groups of confidence reactions are assigned as follows:

$$c_k^{CA/HT} = \begin{cases} \frac{1}{4}, k \in \text{high confidence} \\ \frac{1}{2}, k \in \text{medium confidence} \\ \frac{3}{4}, k \in \text{negativec confidence} \\ 1, k \in \text{other confidence or non-gene-expression} \end{cases} \tag{S2}$$

$v_{f/b,i}^{LB,TR}$ and $v_{f/b,i}^{UB,TR}$ in Eq.(S1) denote the lower and upper bound of the regulated forward-backward fluxes depended on gene- or metabolite-centric approach for activation. The regulation bounds for the gene-centric approach can be expressed as follows:

$$\begin{aligned}
&\text{Regulated bounds for } z_i\text{-th active gene/enzyme:} \\
&\text{Up-regulation:} \\
&\begin{cases} (1-\delta)v_{f,i}^{basal} + \delta v_{f,i}^{UB} \le v_{f,i} \le v_{f,i}^{UB} \\ v_{b,i}^{LB} \le v_{b,i} \le (1-\delta)v_{b,i}^{basal} + \delta v_{b,i}^{LB};\ z_i \in \Omega^{TR} \end{cases} \\
&\text{Down-regulation:} \\
&\begin{cases} v_{f,i}^{LB} \le v_{f,i} \le (1-\delta)v_{f,i}^{basal} + \delta v_{f,i}^{LB} \\ (1-\delta)v_{b,i}^{basal} + \delta v_{b,i}^{UB} \le v_{b,i} \le v_{b,i}^{UB};\ z_i \in \Omega^{TR} \backslash \Omega^{IZ} \end{cases} \\
&\begin{cases} (1-\varepsilon)v_{f,i}^{basal} \le v_{f,i} \le (1+\varepsilon)v_{f,i}^{basal} \\ (1-\varepsilon)v_{b,i}^{basal} \le v_{b,i} \le (1+\varepsilon)v_{b,i}^{basal};\ z_i \in \Omega^{TR} \cap \Omega^{IZ} \end{cases} \\
&\text{Knockout:} \\
&\begin{cases} v_{f,i} = 0 \\ v_{b,i} = 0;\ z_i \in \Omega^{TR} \backslash \Omega^{IZ} \end{cases} \\
&\begin{cases} (1-\varepsilon)v_{f,i}^{basal} \le v_{f,i} \le (1+\varepsilon)v_{f,i}^{basal} \\ (1-\varepsilon)v_{b,i}^{basal} \le v_{b,i} \le (1+\varepsilon)v_{b,i}^{basal};\ z_i \in \Omega^{TR} \cap \Omega^{IZ} \end{cases}
\end{aligned} \tag{S3}$$

where $v_{f,i}^{basal}$ and $v_{b,i}^{basal}$ are the basal value of the ith forward-backward flux obtained from CA and HT templates; Ω^{IZ} is the set of reactions regulated by isozymes determined using the GPR associations, and δ is the modulation parameter determined by a nested hybrid differential evolution (NHDE) algorithm [1]. A reaction catalyzed by isozymes remains around its basal level; thus, we set the flux ratio ε to 0.03 in this study to restrict the flux value. Metabolite-centric regulators modulate the synthesis reactions of the active metabolites. The LBs and UBs of modulated reactions for the ith active metabolite are restricted as follows:

Regulated bounds for the z_i-th active metabolite:

$$\text{Up-regulation:}\begin{cases}(1-\delta)v_{f,j}^{basal}+\delta v_{f,j}^{UB}\le v_{f,j}\le\ v_{f,j}^{UB}\,;j\in N_{ij}>0\text{ and }j\in\Omega^{rxn}\\(1-\delta)v_{b,j}^{basal}+\delta v_{b,j}^{UB}\le v_{b,j}\le\ v_{b,j}^{UB}\,;j\in N_{ij}<0\text{ and }j\in\Omega^{rev}\end{cases}$$

Down-regulation:

$$\begin{cases}v_{f,j}^{LB}\le v_{f,j}\le(1-\delta)v_{f,j}^{basal}+\delta v_{f,j}^{LB}\,;j\in N_{ij}>0\text{ and }j\in\Omega^{rxn}\\v_{b,j}^{LB}\le v_{b,j}\le(1-\delta)v_{b,j}^{basal}+\delta v_{b,j}^{LB}\,;j\in N_{ij}<0\text{ and }j\in\Omega^{rev}\end{cases}$$

Knockout:

$$\begin{cases}v_{f,j}=0\,;j\in N_{ij}>0\text{ and }j\in\Omega^{rxn}\\v_{b,j}=0\,;\ \ j\in N_{ij}<0\text{ and }j\in\Omega^{rev}\end{cases}\qquad\text{(S4)}$$

where N_{ij} is the stoichiometric coefficient of the ith metabolite and the jth reaction.

The computational procedures for calculating CA and HT templates are illustrated in Figure S1.

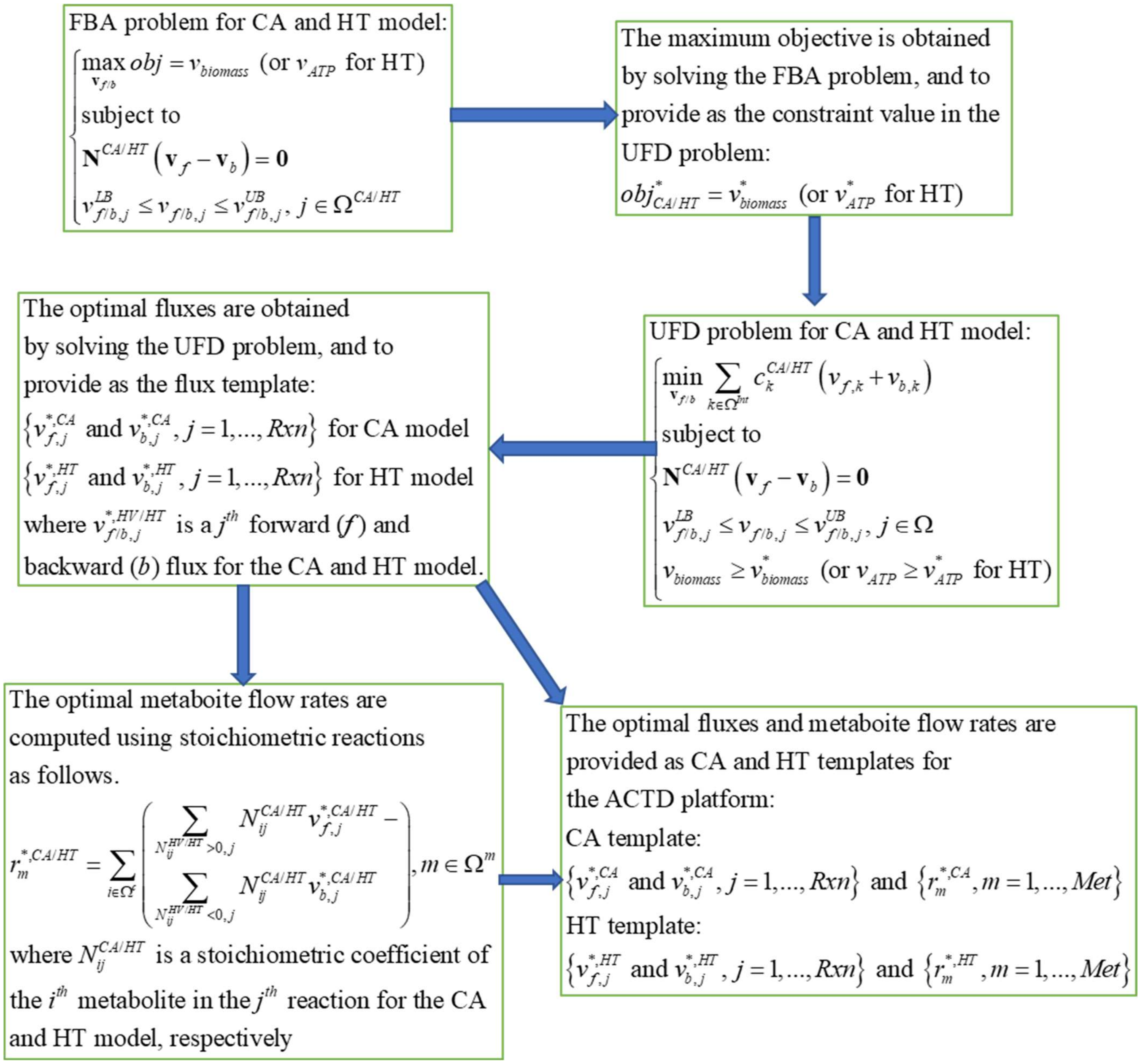

Figure S1. Computational procedures to obtain the optimal fluxes and metabolite flow rates provided as CA and HT templates.

The four goals in the outer optimization problem in Eq.(S1) are explained as follows. The first goal is to evaluate fuzzy minimization ($\widetilde{\min}$) of the biomass growth rate and ATP production rate for treated CA cells (denoted as TR) and the biomass growth rate for perturbed HT cells (denoted as PH) as follows:

$$\widetilde{\min_{\mathbf{z}}}\, v_{biomass}^{TR} \approx 0, \widetilde{\min_{\mathbf{z}}}\, v_{ATP}^{TR} \approx 0, \widetilde{\min_{\mathbf{z}}}\, v_{biomass}^{PH} \approx 0 \qquad \text{(S5)}$$

The second goal is to evaluate fuzzy maximization ($\widetilde{\max}$) of ATP production rate for PH cells as follows:

$$\widetilde{\max_{\mathbf{z}}}\, v_{ATP}^{PH} \approx v_{ATP}^{\max} \tag{S6}$$

The third goal is to measure fuzzy dissimilarity ($\widetilde{\text{dissimilar}}$) that is used to evaluate the disparity of the fluxes and metabolite flow rates for TR and PH cells relative to those of the CA template, as expressed as follows:

$$\begin{cases} \widetilde{\text{dissimilar}}_{\mathbf{z}}\, v_j^{TR} \not\approx v_j^{CA} \\ \widetilde{\text{dissimilar}}_{\mathbf{z}}\, r_m^{TR} \not\approx r_m^{CA} \\ \widetilde{\text{dissimilar}}_{\mathbf{z}}\, v_j^{PH} \not\approx v_j^{CA} \\ \widetilde{\text{dissimilar}}_{\mathbf{z}}\, r_m^{PH} \not\approx r_m^{CA} \end{cases} \tag{S7}$$

The four goal is to measure fuzzy similarity ($\widetilde{\text{similar}}$) of fluxes ($v_j$) and metabolite flow rates (r_m) of TR and PH cells relative to the healthy (HT) template as follows:

$$\begin{cases} \widetilde{\text{similar}}_{\mathbf{z}}\, v_j^{TR} \approx v_j^{HT} \\ \widetilde{\text{similar}}_{\mathbf{z}}\, r_m^{TR} \approx r_m^{HT} \\ \widetilde{\text{similar}}_{\mathbf{z}}\, v_j^{PH} \approx v_j^{HT} \\ \widetilde{\text{similar}}_{\mathbf{z}}\, r_m^{PH} \approx r_m^{HT} \end{cases} \tag{S8}$$

In the aforementioned equations, the decision vector **z** represents the gene encoding enzymes as determined by a nest hybrid differential evolution (NHDE) algorithm (Described in the next section) for modulation. The fluxes $v_j^{CA/HT}$ and metabolite flow rates $r_m^{CA/HT}$ of the CA and HT templates can be obtained from clinical experimental data (if available). However, genome-scale clinical data are currently not available. Both templates computed from the CA and HT models as discussed in Figure S2 are provided for the computation. The mth metabolite flow rate is computed using the following equations:

$$r_m = \sum_{i \in \Omega^c} \left(\sum_{N_{ij} > 0, j} N_{ij} v_{f,j} - \sum_{N_{ij} < 0, j} N_{ij} v_{b,j} \right), m \in \Omega^m \tag{S9}$$

where Ω^c is the set of species located in various compartments of CA and HT cells, and N_{ij} is a stoichiometric coefficient of the ith metabolite in the jth reaction of each GSMM. The forward flux $v_{f,j}$ and backward flux $v_{b,j}$ of the jth reaction are calculated by applying FBA and UFD models in the inner optimization problem as described in Eq.(S1).

The ACTD problem in Eq.(S1) is transformed into a maximizing decision-making (MDM) problem through fuzzy set theory as illustrated in Figure 3 of the main text. The MDM problem is expressed as follows:

$$\begin{cases} \max_{\mathbf{z}} \eta_D = \max_{\mathbf{z}} \left(\eta_{CV}^{TR} + \min\left\{ \eta_{CV}^{TR}, \eta_{CV}^{PH}, \eta_{MD}^{TP} \right\} \right) / 2 \\ \text{subject to inner optimization problems} \\ \text{1. FBA and UFD problems for treated CA cells} \\ \text{2. FBA and UFD problems for perturbed HT cells} \end{cases} \quad \text{(S10)}$$

where η_{CV}^{TR}, η_{CV}^{PH} and η_{MD}^{TP} denote as the cell viability grade of the TR model, cell viability grade of the PH model and metabolic deviation grade of the TR and PH models relative to their corresponding templates, and define as follows:

$$\eta_{CV}^{TR} = \left(\eta_{biomass}^{TR} + \min\left\{ \eta_{biomass}^{TR}, \eta_{ATP}^{TR} \right\} \right) / 2 \quad \text{(S11)}$$

$$\eta_{CV}^{PH} = \eta_{ATP}^{PH} \quad \text{(S12)}$$

$$\eta_{MD}^{TP} = \frac{1}{2}\left(\frac{\left(\eta_{MD}^{TRHT} + \eta_{MD}^{PHHT} + \eta_{MD}^{TRCA} + \eta_{MD}^{PHCA} \right)}{4} + \min\left\{ \eta_{MD}^{TRHT}, \eta_{MD}^{PHHT}, \eta_{MD}^{TRCA}, \eta_{MD}^{PHCA} \right\} \right) \quad \text{(S12)}$$

The membership functions, $\eta_{biomass}^{TR}$ and η_{ATP}^{TR}, in Eq.(S11) use to represent fuzzy minimization and maximization for the TR model in Eqs.(S5) and (S6). Similarly, the membership function, η_{ATP}^{PH}, attribute to fuzzy optimization for the PH model in Eqs.(S5) and (S6). The membership grades for fuzzy minimization and fuzzy maximization are expressed in Figure S3. Both fluxes and metabolite flow rates for TR and PH models are used to compute the corresponding metabolic deviation grades through two-sided membership functions (Figure S2). The grades are then used to compute overall metabolic deviation grades of fuzzy similarity (η_{MD}^{TRHT} and η_{MD}^{PHHT}) relative to the HT template and fuzzy dissimilarity (η_{MD}^{TRCA} and η_{MD}^{PHCA}) relative to the CA template, respectively.

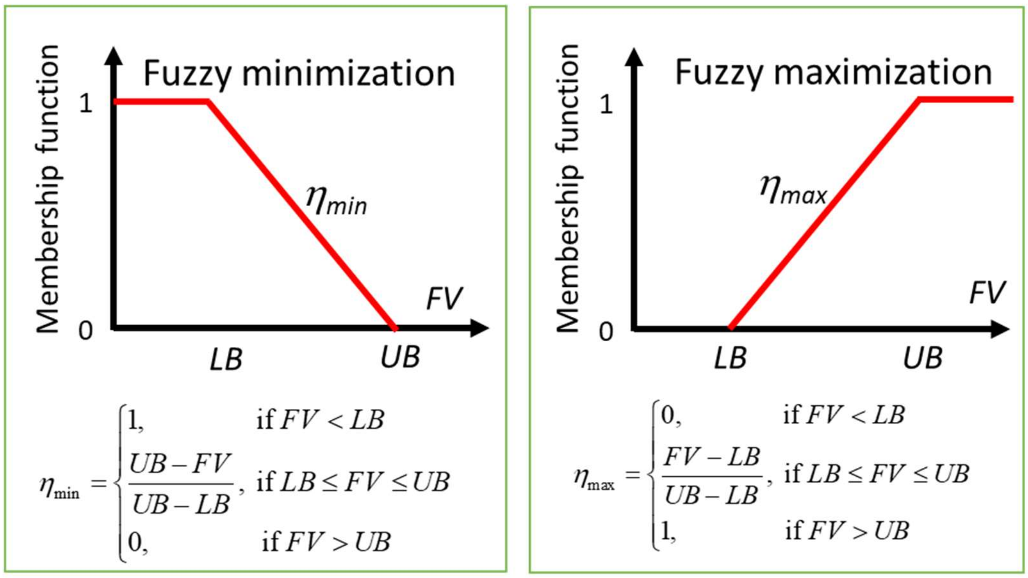

$$\eta_{\min} = \begin{cases} 1, & \text{if } FV < LB \\ \dfrac{UB - FV}{UB - LB}, & \text{if } LB \le FV \le UB \\ 0, & \text{if } FV > UB \end{cases}$$

$$\eta_{\max} = \begin{cases} 0, & \text{if } FV < LB \\ \dfrac{FV - LB}{UB - LB}, & \text{if } LB \le FV \le UB \\ 1, & \text{if } FV > UB \end{cases}$$

The flux *FV* is obtained by solving Eq.(S1), and the lower bound *LB* and the upper bound *UB* are provided from HV and HT templates from Figure S2.

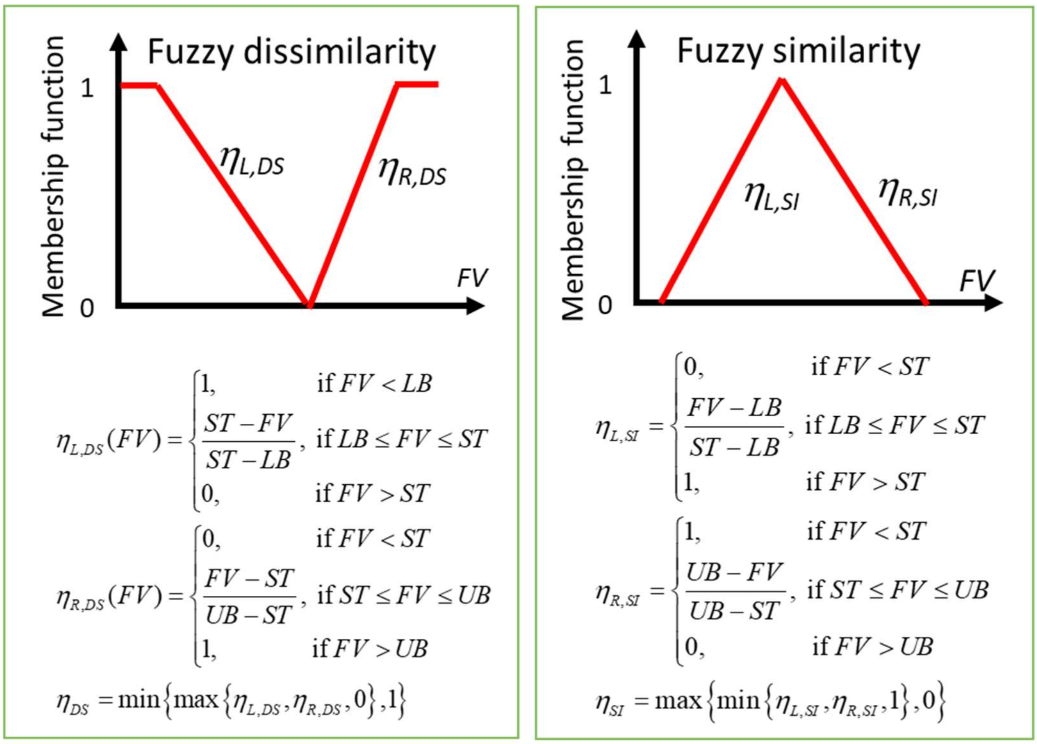

$$\eta_{L,DS}(FV) = \begin{cases} 1, & \text{if } FV < LB \\ \dfrac{ST - FV}{ST - LB}, & \text{if } LB \le FV \le ST \\ 0, & \text{if } FV > ST \end{cases}$$

$$\eta_{R,DS}(FV) = \begin{cases} 0, & \text{if } FV < ST \\ \dfrac{FV - ST}{UB - ST}, & \text{if } ST \le FV \le UB \\ 1, & \text{if } FV > UB \end{cases}$$

$$\eta_{DS} = \min\left\{\max\left\{\eta_{L,DS}, \eta_{R,DS}, 0\right\}, 1\right\}$$

$$\eta_{L,SI} = \begin{cases} 0, & \text{if } FV < ST \\ \dfrac{FV - LB}{ST - LB}, & \text{if } LB \le FV \le ST \\ 1, & \text{if } FV > ST \end{cases}$$

$$\eta_{R,SI} = \begin{cases} 1, & \text{if } FV < ST \\ \dfrac{UB - FV}{UB - ST}, & \text{if } ST \le FV \le UB \\ 0, & \text{if } FV > UB \end{cases}$$

$$\eta_{SI} = \max\left\{\min\left\{\eta_{L,SI}, \eta_{R,SI}, 1\right\}, 0\right\}$$

FV denotes as a flux of ith reaction or a metabolite flow rate of ith metabolite that is obtained by solving Eq.(S1), and ST denotes as the optimal value of HV or HT templates obtained from Figure S2. The lower bound LB and the upper bound UB are provided from HV and HT templates as follows: LB = ST/4 and UB = 4ST.

Figure S2. Evaluation of membership grades for fuzzy minimization, fuzzy maximization, fuzzy similarity and fuzzy dissimilarity.

Based on fuzzy set theory, the FMHO problem in Eq.(S1) can be transformed into a maximizing decision-making (MDM) problem in Eq.(S10). The MDM problem can be solved using the nested hybrid differential evolution (NHDE) algorithm [1]. The optimality conditions of this transformation were proved in a previous study. According to optimality conditions, a Pareto solution to the FMHO problem can be derived based on the transformed MDM problem. MDM problems are challenging optimization problems that cannot be solved directly using commercially available software. The high-dimensional, bilevel, and mixed-integer linear characteristics of these problems make them NP-hard.

In this study, we applied the NHDE algorithm, which is a stochastic and parallel direct search algorithm based on procedures in hybrid differential evolution (HDE) [2], an extension of the original differential evolution algorithm [3]. The computational procedures of the NHDE algorithm are detailed in Table 1. The initialization process involves randomly generating a population of Np individuals (z_i) to cover the entire search space uniformly. A structure array is used to represent the order number of a candidate target, its corresponding regulation, and the regulated strength parameter. Additionally, a two-group strategy was established to represent candidate targets and identify combinations of candidate genes and nutrient uptakes, as illustrated in Figure 4 of the main text.

Introduction to Nested Hybrid Differential Evolution (NHDE)

The ACTD platform is formulated as a fuzzy multi-objective hierarchical optimization problem, that can be transformed into a maximizing decision-making (MDM) problem by using fuzzy set theory to derive Pareto solutions as shown in Eq.(S10). The existence and limitation of the transformation have proved in Wang, et al. [1]. The MDM problem is rewritten as the following simplified formulation for easily explaining the NHDE algorithm.

$$
\begin{cases}
\text{Outer optimization problem:} \\
\quad \max f(\mathbf{x},\mathbf{z}) \\
\text{subject to the inner optimization problems:} \\
\begin{cases}
\text{FBA problem} \\
\max\limits_{\mathbf{x}} obj = \mathbf{c}^T \mathbf{x} \\
\text{subject to} \\
\quad \mathbf{A}\mathbf{x} = \mathbf{0} \\
\quad \mathbf{x}_{LB} \le \mathbf{x} \le \mathbf{x}_{UB},\ \mathbf{z} \notin \Omega^{TR} \\
\quad \mathbf{x}_{LB}^{TR} \le \mathbf{x} \le \mathbf{x}_{UB}^{TR},\ \mathbf{z} \in \Omega^{TR} \\
\quad \mathbf{x} \ge \mathbf{0}
\end{cases}
\begin{cases}
\text{UFD problem} \\
\min\limits_{\mathbf{x}} \sum c_k x_k \\
\text{subject to} \\
\quad \mathbf{A}\mathbf{x} = \mathbf{0} \\
\quad \mathbf{x}_{LB} \le \mathbf{x} \le \mathbf{x}_{UB},\ \mathbf{z} \notin \Omega^{TR} \\
\quad \mathbf{x}_{LB}^{TR} \le \mathbf{x} \le \mathbf{x}_{UB}^{TR},\ \mathbf{z} \in \Omega^{TR} \\
\quad \mathbf{c}^T \mathbf{x} \ge \mathbf{c}^T \mathbf{x}^* \\
\quad \mathbf{x} \ge \mathbf{0}
\end{cases}
\end{cases}
\tag{S13}
$$

The inner optimization problem consists of two linear programming problems, which is a sequential relationship.

The NHDE algorithm is a stochastic optimization based on hybrid differential evolution [2], which was extended from the original DE algorithm [3]. The basic operations of original DE and modified NHDE are shown in Table S1.

Table S1. Basic operations for the original DE and NHDE algorithms

Original DE	NHDE
1. Representation and initialization	1. Representation and initialization
2. Mutation	2. Mutation with rounding operation
3. Crossover operation	3. Crossover operation
4. Selection and evaluation	4. Restriction operation
5. Repeat steps 2 to 4	5. Selection and evaluation
	6. Solve LP/QP problems for each candidate gene
	7. Compute fitness for each feasible design
	8. Migration operation performed naturally or enforced if necessary
	9. Repeat steps 2 to 6

The computational procedures of NHDE are listed in Table 1 of the main text. NHDE is a parallel direct search algorithm (as shown in Figure S3) that utilizes a population of N_p individuals (enzymes and exchange reactions) to find an optimal solution. The initialization process randomly generates N_p individuals to cover the entire search space uniformly. Each individual in the population consists of a set of pair of enzymes and exchange reactions that are selected to be modulated.

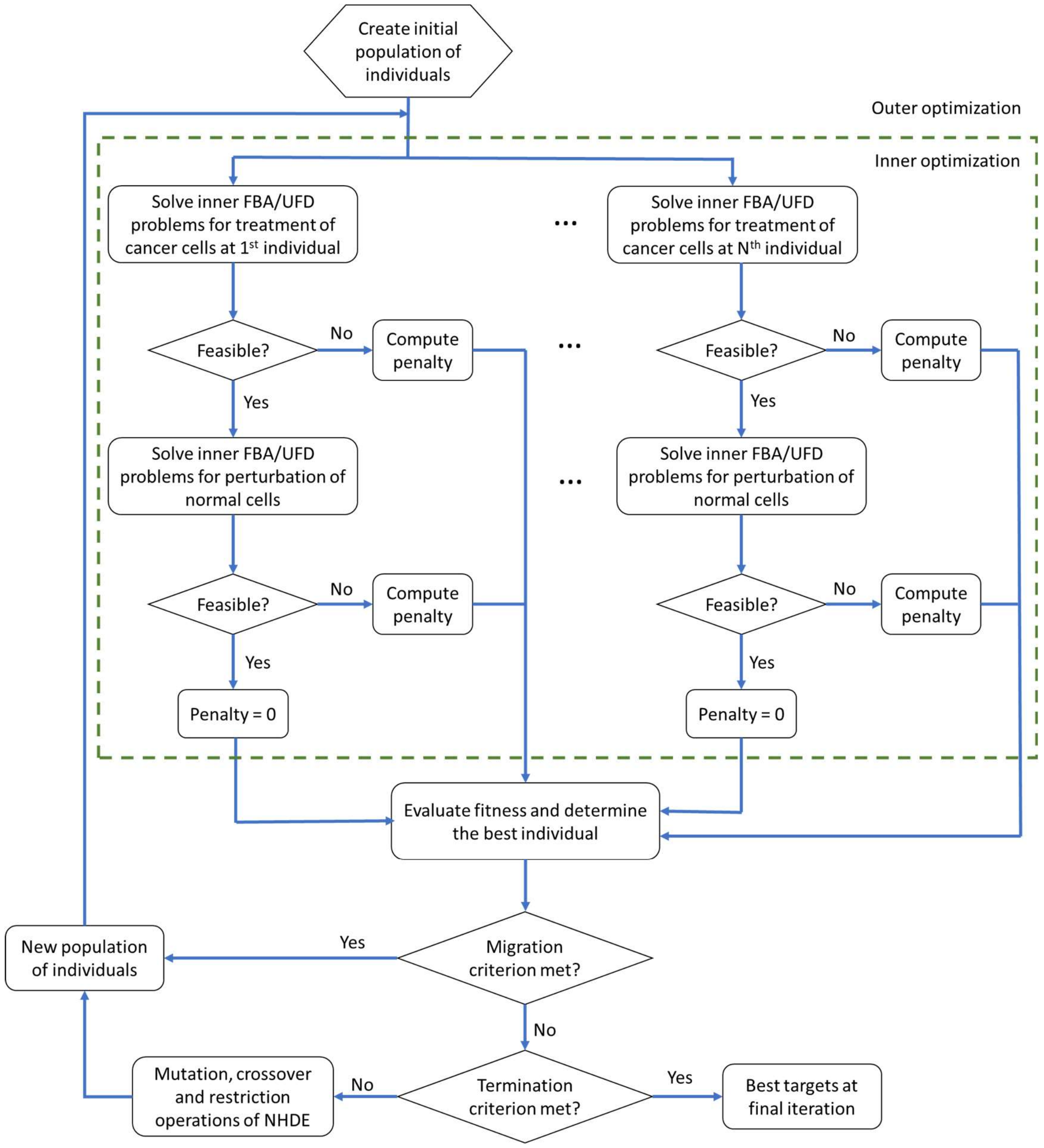

Figure S3. Flowchart of the parallel search algorithm in NHDE

The operations in Table S1 of the NHDE algorithm are explained as follows. The mutation operator of NHDE adopted from DE was an essential component compared with other evolutionary algorithms. Different from conventional evolutionary algorithms, the mutation operation of DE/NHDE uses the difference between two or four randomly chosen individuals as an evolutionary direction. The ith mutant individual $(\mathbf{z}^G)_i$ in generation G is obtained through the difference of two or four random individuals as expressed in the following form:

$$(\mathbf{z}^G)_i = \mathrm{INT}\left\{(\mathbf{z}^G)_p + \rho^G\left[(\mathbf{z}^G)_j - (\mathbf{z}^G)_k + (\mathbf{z}^G)_l - (\mathbf{z}^G)_m\right]\right\}, i = 1,\ldots,N_p \quad \text{(S14)}$$

where random indices $j,k,l,m \in \{1,\ldots,N_p\}$ are mutually different. The operator INT in the equation is used to rounding the real vector into the integer vector. In DE, the differential mutation factor $\rho^G \in [0, 1]$ is fixed and set by the user to obtain faster convergence. This factor is used to control the step length along the searching direction. A random mutation factor was used in NHDE to obtain more diversified individuals. NHDE also includes an additional mutation strategy that applying a linear crossover for the ith individual and the best individual $(\mathbf{z}^G)_b$ to generate the parent individual. The parent individual is therefore expressed as follows:

$$(\mathbf{z}^G)_p = \rho_p^G(\mathbf{z}^G)_b + (1-\rho_p^G)(\mathbf{z}^{G-1})_i \tag{S15}$$

where the factor $\rho_p{}^G$ is a random number between zero and one generated by a uniform distribution generator, and $(\mathbf{z}^{G-1})_i$ indicates the ith mutant individual in the previous generation. The mutation operation may cause the mutant individual escape from the search domain. The mutation operation may cause the mutant individual to escape the search domain (i.e., bounds are violated). If this occurs, it is replaced by a random number within the lower and upper bounds of the particular decision variable, thus restricting to the search domain. The choice of mutation factor for DE/NHDE is heuristic and random. When population diversity is low, candidate individuals rapidly cluster together such that the individuals cannot be further improved, and premature convergence occurs. Similar to conventional evolutionary algorithms, the local population diversity could be increased by using a crossover operation such as a binomial crossover.

NHDE use the difference between two or four mutually independent individuals to determine the direction of search and obtain a mutant individual. This differential mutation converges quickly so that most individuals cluster around the best candidate individual in some generations. Consequently, the population diversity and exploration capability diminish and clustered individuals are unable to reproduce more diversified individuals through the mutation operation because the weighted difference is nearly zero. The recombination of mutant individuals and their clustered parents further prevents the reproduction of a diversified population. Therefore, all individuals quickly cluster together and superior individuals cannot be generated through mutation and crossover operations.

The migration operation of the NHDE algorithm is used to help individuals escape from the local cluster, but this operation is performed only if the population diversity falls below a desired level. The degree of population diversity ζ is introduced to check whether the migration operation should be performed. Each element of the ith individual $(\mathbf{z}^G)_i$ in generation G is referred to as a gene of the individual, and the gene diversity index dz_{ji} is given by

$$dz_{ji} = \begin{cases} 0, & \text{if } z_{ji}^G = z_{jb}^G,\ j = 1,...,n; i = 1,...,N_p; i \neq b \\ 1, & \text{otherwise,} \end{cases} \quad \text{(S16)}$$

where z_{ji}^G and z_{jb}^G are the jth gene of the ith and best individual at the Gth generation, respectively. dz_{ji} is set to zero if the jth gene of the ith individual is identical to the best gene; otherwise it is set to one (10). ζ is defined as the ratio of total gene diversities to the total number of genes other than those of the best individual:

$$\zeta = \frac{\sum_{i=1, i\neq b}^{N_p} \sum_{j=1}^{n} dz_{ji}}{n(N_p - 1)} \quad \text{(S17)}$$

The value of population diversity degree ranges between zero and one. A value of zero implies that all of the genes are clustered around the best individual. On the other hand, a value of one indicates that current candidate individuals are a completely diversified population. The desired tolerance for population diversity is assigned by the user. A tolerance value of zero implies that the migration operation in NHDE is switched off, and one implies that the migration operation is performed at every generation. Consequently, the user can set a tolerance value for population diversity degree, $\varepsilon \in (0, 1)$. If ζ is smaller than ε, then NHDE performs migration operations to regenerate a new population in order to escape from a local point; otherwise, NHDE suspends the migration operation and maintains a constant search direction toward finding a new solution.

References

1. F.S. Wang, T.Y. Wang, W.H Wu, Fuzzy multiobjective hierarchical optimization with application to identify antienzymes of colon cancer cells, Journal of the Taiwan Institute of Chemical Engineers. 2022; 132:10412. http://doi.org/10.1016/j.jtice.2021.10.021
2. J.P. Chiou, F.S. Wang, Hybrid method of evolutionary algorithms for static and dynamic optimization problems with application to a fed-batch fermentation process, Computers & Chemical Engineering. 1999; 23:1277-1291. http://doi.org/10.1016/S0098-1354(99)00290-2
3. R. Storn, K. Price, Differential evolution - A simple and efficient heuristic for global optimization over continuous spaces, Journal of Global Optimization. 1997; 11:341-359. http://doi.org/10.1023/A:1008202821328
